# Transcriptional profiling of glutathione metabolism and the glutathione redox cycle of *Festuca sinensis* infected with *Epichloë sinensis* in response to Na_2_SeO_3_

**DOI:** 10.3389/fpls.2026.1752324

**Published:** 2026-03-19

**Authors:** Lianyu Zhou, Yun Ma, Yu Liu, Qiaoyu Luo, Feng Qiao, Huichun Xie, Duocheng Sang, Wenyu Ma

**Affiliations:** 1 School of Life Science, Qinghai Normal University, Xining, China; 2 Qinghai Normal University, Key Laboratory of Medicinal Plant and Animal Resources of the Qinghai-Tibetan Plateau in Qinghai Province, Xining, China; 3 Qinghai Normal University, Academy of Plateau Science and Sustainability, Xining, China

**Keywords:** differentially expressed genes, *Epichloë* endophyte, *Festuca sinensis*, glutathione metabolism, selenium

## Abstract

*Epichloë* endophytes enhance tolerance to metal stress in various cool-season grasses, but the mechanism of *Epichloë*-associated grasses responding to different concentrations of Na_2_SeO_3_ are still unclear. After *Festuca sinensis* infected with (EI) or without (EF) *Epichloë sinensis* were grown for 90 days, the physiochemical and transcriptional profiling of glutathione metabolism in both shoots and roots over a period of 3 days was investigated in pot trials supplied with 0, 20, and 50 mg/L Na_2_SeO_3_ using high-throughput RNA sequencing technology. Results of the study showed that *Epichloë* endophytes increased the glutathione reductase (GR) levels in shoots and roots and reduced the glutathione (GSH) and GSH/GSSG ratio in shoots. Treatment with 20 mg/L Na_2_SeO_3_ resulted in elevated levels of GR and GSSG in both shoots and roots of EI and EF plants. In line with the accumulation of GSH in plant tissues, the endophyte enhanced the expression of *GCLC* (glutamate-cysteine ligase catalytic subunit), *GR*, *APX* (ascorbate peroxidase), and 5*-OPase* (5-oxoprolinase) in shoots in response to 20 mg/L Se, and *Grx* (glutaredoxin) on day 2 in the presence of Se, while reducing most *GST* (glutathione S-transferase) genes. Under selenium treatment, the expression of most *GST* and *GR* genes was upregulated in shoots of both EI and EF plants, with a higher accumulation of *GST* genes observed in roots. Our findings suggest that the *Epichloë* endophyte differentially activated plant glutathione metabolism depending on treatment duration under Se application and could represent a promising strategy for enhancing Se tolerance in *F. sinensis*.

## 1 Introduction

Selenium (Se) is a crucial component of glutathione peroxidase (GPX) in animals. At appropriate concentrations, it enhances plant nutritional quality, promotes growth and development (Quinn et al., 2011; Banerjee and Roychoudhury, 2019), increases resistance to stress (Cartes et al., 2005; Chen et al., 2008; Feng et al., 2013), improves the concentration of a certain compound (Ghasemian et al., 2021), and alleviates metal toxicity (Cartes et al., 2010; Cai et al., 2019; Li et al., 2020). Se exists in a variety of oxidation states (−2, 0, + 4, and +6), and certain plants metabolize it. Selenite (+4) is first reduced to selenide (−2) by sulfite reductase or reduced glutathione (GSH). Subsequently, the conversion of selenide to selenocysteine (SeCys) is facilitated by O-acetylserine thiol lyase (OAS-TL) and O-acetylserine (OAS). Selenocysteine is further transformed into various forms of Se through different metabolic pathways (Terry et al., 2000; Sors et al., 2005; Valassakis et al., 2018). In addition to Se metabolites, plants respond to Se stress by modulating GSH concentrations through GSH metabolism (Jiang et al., 2016; Freeman et al., 2010). For instance, Se treatment reduces GSH levels in plants and is accompanied by an increase in the sulfate transporter (S*ULTR1;1*) (Takahashi et al., 2000; Cardoso et al., 2023). The disruption of glutathione biosynthesis by selenate leads to decreased GSH levels (Grant et al., 2011), indicating that GSH plays a crucial role in conferring resistance to selenate toxicity in plants (Hugouvieux et al., 2009). When sodium selenite treatment ranged from 0.5 to 20 mg/kg, both GPX and GSH levels increase in rice leaves (Dai et al., 2019). Thus, GSH can act as a non-enzymatic antioxidant, and it is proposed that selenite-induced GSH mitigates a selenite-defensive response.

Glutathione, primarily in its reduced form (GSH), is converted to oxidized glutathione (GSSG) by the enzyme GPX. The conversion of GSSG back to GSH is facilitated by glutathione reductase (GR), resulting in variations in the GSH/GSSG ratio. GSH metabolism in plants encompasses both synthesis and degradation processes. Its synthesis involves γ-glutamylcysteine synthetase (GSH1) and glutathione synthetase (GSH2), and its breakdown is mostly catalyzed by gamma-glutamyl cyclotransferases (GGCTs) and 5-oxoprolinase (5-OPase). According to reports, γ-glutamyl transpeptidases (γ-GT) and glutaredoxin (Grx) play a vital role in GSH homeostasis (Chaudhary et al., 2024; Paulose et al., 2013). Previous studies have suggested that the assimilation and tolerance of Se by plants are influenced by genes and proteins associated with GSH metabolism (Chauhan et al., 2020; Zhang et al., 2019). The cytosolic ascorbate peroxidase gene (*APX1*)-mediated Se tolerance has been linked, at least in part, to the enhanced activities of antioxidant enzymes, including catalase (CAT), GPX, and GR (Jiang et al., 2016). The expression level of the selenocysteine methyltransferase (*SMT*) gene in kidney beans increases under Se supplementation (Patel et al., 2021). Furthermore, the expression of the *APX* and *GST* genes involved in GSH metabolism is upregulated in the roots of *Camellia sinensis* supplemented with Na_2_SeO_3_ (Ren et al., 2022).

The tolerance of selenium in plants is not only influenced by exogenous Se levels and plant species but also closely linked to microorganisms (Freeman et al., 2010). Numerous bacteria and fungi are now recognized for their capabilities in selenium transport, reduction, oxidation, assimilation, and methylation (Kessi and Hanselmann, 2004; Rosenfeld et al., 2017; Staicu et al., 2015; Wang et al., 2022). Microbes associated with plants have been shown to affect Se bioavailability (Munier-Lamy et al., 2007), as well as plant Se accumulation and volatilization (Sura-De Jong et al., 2015; Kaur et al., 2022; De Souza et al., 1999; Durán et al., 2013, Durán et al., 2018; Lindblom et al., 2018), speciation, and distribution (Yu et al., 2011; Chen et al., 2020). Endophytic selenobacteria inoculated in lettuce plants significantly increases the activities of APX, CAT, and GR compared with mycorrhizal fungus (Durán et al., 2016). Additionally, arbuscular mycorrhizal (AM) symbiosis may influence Se acquisition and toxicity by altering the expression of sulfate and inorganic phosphate transporter genes (Li et al., 2022; Sun et al., 2021; Luo et al., 2019). Previous transcriptome analyses indicated that differentially expressed genes (DEGs) in the comparisons of control *vs*. Se, AM *vs*. AM+Se, and Se *vs*. Se+AM are enriched in glutathione metabolism (ko00480) (Qin et al., 2023). However, few studies have examined the expression of plant genes such as *GCLC*, *GR*, *GST*, *GGT*, *GGCT*, *APX*, *Grx*, *5-OPase*, and *6PGD*, which are also associated with glutathione metabolism in endophyte-infected plants in response to Se.

*Epichloë* endophytes infect pooid grass genera worldwide, enhancing the grasses’ resistance to both abiotic and biotic stresses. The benefits of *Epichloë* endophytes for *Festuca sinensis* include increased tolerance to cold (Zhou et al., 2021), drought (Xu et al., 2021), waterlogging (Wang et al., 2017), and heavy metals (Wang et al., 2021a), as well as protection against pathogens (Wang et al., 2021b) and mitigation of reverse degradation (Yao et al., 2015). *Epichloë* endophytes regulate host growth; enhance the activities of SOD, CAT, peroxidase, and APX enzymes; and adjust hormone and alkaloid concentrations, thereby improving the plant’s tolerance to mineral imbalance stress (Wang et al., 2021a; Bonnet et al., 2000; Zhang et al., 2010; Malinowski and Belesky, 2019). However, only a limited amount of research has explored the mechanisms by which *Epichloë*-associated *F. sinensis* responds to selenium stress using high-throughput RNA sequencing technology. The experimental hypothesis of the present study was that the *Epichloë* endophyte could modulate the grass shoot or root response to Se by changing glutathione metabolizing genes. To test this hypothesis, we established *F. sinensis* infected with (EI) and without (EF) *Epichloë sinensis* and then set a challenge of three concentrations of Na_2_SeO_3_. The concentrations of GSH and GSSG, the activity of GR, and the expression levels of genes associated with glutathione metabolism were investigated in the shoots and roots of the grass. This study may provide novel insights into the modulation of the glutathione redox cycle and associated gene networks, offering valuable contributions to research on plant–microbe interactions and abiotic stress.

## 2 Materials and methods

### 2.1 Plant material and experimental treatments

The taxonomic identification of *F. sinensis* was determined using taxonomic keys (Ma and Xu, 2012). *F. sinensis* seeds were collected, stained with aniline blue, and examined by microscopy (Olympus, Japan) in 2012. These plants were infected with the *Epichloë* endophyte (Tian et al., 2020). To obtain seeds free of *Epichloë sinensis*, some seeds were treated for 8 h with the fungicide thiophanate-methyl (300× dilution) (Yao et al., 2013). Fungicide-treated seeds and untreated seeds were sown separately in the experimental field of the College of Pastoral Agriculture Science and Technology, Yuzhong Campus of Lanzhou University (104°39'E, 35°89'N, altitude 1,653 m) (Zhou et al., 2021). The infection status of each plant was checked by microscopy on aniline blue-stained leaf sheath pieces. Seeds were collected from *Epichloë* endophyte-infected (EI) and endophyte-free (EF) *F. sinensis* plants. The voucher specimens of plant species used in this study are deposited in the Key Laboratory of Medicinal Plant and Animal Resources of the Qinghai-Tibetan Plateau, Qinghai Normal University, Xining. In our initial studies, Se concentrations of 0, 2, 20, and 50 mg/L had no significant effect on the number of tillers or root length of plants grown for 6 weeks; only the 20 mg/L Se treatment increased plant height. The *Epichloë* endophyte influenced plant Se accumulation differently at 20 and 50 mg/L Se applications. Therefore, Se was supplied at concentrations of 0 (control), 20, and 50 mg/L in this study. Three EI or EF seeds were planted in a single plastic pot (bottom diameter: 9 cm, top diameter: 16 cm, height: 11 cm) filled with 750.0 g of sterilized medium (soil: river sand, 2: 1, v/v), and later thinned to two seedlings per pot. The pots (45 for EI plants and 45 for EF plants) were incubated in a constant-temperature greenhouse maintained at 22 °C, with a 16-h light and 8-h darkness cycle using 40-W light emitting diode (LED) lighting. The plants were watered with distilled water as needed. After 90 days, when the plants had approximately 9–11 tillers, they were irrigated with 100 mL of Na_2_SeO_3_ solution at three concentrations: 0, 20, and 50 mg/L. For each Se treatment, there were 30 pots for both EI and EF plants. The shoots and roots from five randomly selected pots of each treatment (10 plants) were harvested separately on days 1, 2, and 3, immediately frozen in liquid nitrogen, and stored in a freezer at −80 °C for subsequent analysis of glutathione content and transcriptome profiling.

### 2.2 Determination of the glutathione redox cycle

100 mg of plant tissues (either shoots or roots) was macerated in 5 mL of potassium phosphate buffer (0.06 mol/L). The resulting extract was then centrifuged for 15 min at 20,000 × g. The supernatant was used to estimate GR activity, as well as the concentrations of GSSG and total glutathione (total-GSH), following the protocols provided by the assay kits (Shanghai Jining Industry Co., Ltd., Shanghai, China, www.shjning.com). The concentration of GSH was determined by subtracting GSSG from total-GSH. Three independent biological replicates were performed for each treatment in this study. The specific kits used were as follows: the GR activity kit with item number JN24288 and product number JN20240527514, GSSG kit with item number JN24282 and product number JN20240527516, and total-GSH kit with item number JN72571A and product number JN20240527515.

### 2.3 RNA isolation, sequencing, and analysis

Plant tissues were utilized to extract total RNA using TRIzol reagent (Invitrogen, Carlsbad, CA, USA) in accordance with the manufacturer’s protocol. RNA-Seq analysis was performed with three replicates. The concentration and integrity of the RNA were assessed using a spectrophotometer (NanoDrop 2000) and an Agilent Bioanalyzer 2100 system (Agilent Technologies, Santa Clara, CA, USA). A total of 1 µg of RNA per sample was used to generate sequencing libraries with the NEBNext^®^ Ultra™ RNA Library Prep Kit for Illumina^®^ (NEB, San Diego, CA, USA). The library fragments were purified using an AMPure XP system (Beckman Coulter, Beverly, CA, USA). Subsequently, the libraries were sequenced on an Illumina NovaSeq X Plus PE150.

For the analysis of gene expression, RSEM software (version 1.2.19) was utilized to determine the read count for each gene, and the fragments per kilobase per million (FPKM) for each gene were calculated based on gene length and mapped read counts. Gene expression comparisons were conducted under various conditions. Differential expression analysis was performed using DESeq2 (version 1.30.1) with a false discovery rate (FDR) of ≤0.01 and a fold change of ≥2.0. Unigene sequences were aligned using DIAMOND (version 2.0.4) (Buchfink et al., 2015) against the following databases: GO (Gene Ontology), KEGG (Kyoto Encyclopedia of Genes and Genomes), Swiss-Prot (a manually annotated and reviewed protein sequence database), and Pfam (Protein family) annotation. The statistical enrichment of differentially expressed genes (DEGs) in the KEGG pathway was assessed using KOBAS software (version 2.0) (Xie et al., 2011). To determine the degree of DEGs changes, Origin software (version 2021) was used to construct a heatmap based on values of log2 fold change (FC).

### 2.4 Data analysis of the glutathione redox cycle

Using SPSS 26 software, we analyzed the differences in GR activity, GSH, and GSSG in relation to the *Epichloë* endophytic status, Se levels, and treatment duration through a three-way analysis of variance (ANOVA). Following the identification of statistically significant results, we conducted simple main effect analyses to evaluate the effects of Se levels or treatment duration for each treatment condition using the Fisher’s least significant difference (LSD) test at *p* < 0.05 with a sample size of 18. All analyses met the assumptions of ANOVA (residual independence, normality, and homogeneity of variance). All values are presented as means ± standard deviation.

## 3 Results

### 3.1 Effects on GR activity

Highly significant effects (*p* < 0.01, **Table 1**) of the *Epichloë* endophyte, Se concentration, and treatment duration were observed on GR activities in the shoots and roots of *F. sinensis*. Additionally, interactions between the *Epichloë* endophyte, Se concentration, and treatment duration were noted for GR activities in both shoots and roots (*p* < 0.05). As illustrated in **Tables 2**, **3**, on day 1 and day 2, the EI shoots exhibited higher GR activity compared with the EF plants treated with Se concentrations (*p* < 0.05). Furthermore, a concentration of 20 mg/L Se significantly enhanced shoot GR activity. On the first day, Se treatment significantly increased root GR activities by 154.84% to 307.07% when compared with the control concentration, whereas the endophyte also elevated root GR activities under control conditions. The EI roots treated with 20 mg/L Se displayed the highest levels of GR activities on the second day among all treatments. On the third day, GR activities in the control and 50 mg/L Se-treated EI shoots were 8.97% to 148.47% higher than those in other treatments (*p* < 0.05), and the GR activities of EI roots under 50 mg/L Se were the highest among all root samples.

**Table 1:** Results of a three-way analysis of variance examining the effects of *Epichloë endophyte* (E), selenium concentration (Se), and treatment duration (T) on glutathione reductase activity, reduced glutathione, and oxidized glutathione in *F. sinensis*.

Glutathione redox cycle	Source		E	Se	T	E×Se	E×T	Se×T	E×Se×T
Glutathione reductase activity (nmol/min/mg protein)	Shoot	F	7.379	9.083	19.692	10.526	4.184	16.216	14.190
		*p*	0.010	0.001	0.000	0.000	0.023	0.000	0.000
	Root	F	24.104	26.921	12.891	4.932	9.634	34.034	3.066
		*p*	0.000	0.000	0.000	0.013	0.000	0.000	0.028
Oxidized glutathione (nmol/g)	Shoot	F	35.853	73.754	155.093	315.061	8.094	66.432	82.701
		*p*	0.000	0.000	0.000	0.000	0.001	0.000	0.000
	Root	F	0.216	178.220	59.713	143.758	107.277	111.317	33.947
		*p*	0.645	0.000	0.000	0.000	0.000	0.000	0.000
Reduced glutathione (nmol/g)	Shoot	F	15.931	14.970	126.315	3.318	1.960	2.489	0.824
		*p*	0.000	0.000	0.000	0.048	0.156	0.060	0.519
	Root	F	0.187	16.917	124.551	1.557	6.345	26.260	0.957
		*p*	0.668	0.000	0.000	0.225	0.004	0.000	0.443
GSH/GSSG	Shoot	F	19.448	26.481	148.600	1.689	2.029	3.918	5.958
		*p*	0.000	0.000	0.000	0.199	0.146	0.010	0.001
	Root	F	0.001	14.808	131.043	3.077	10.553	23.286	1.110
		*p*	0.972	0.000	0.000	0.058	0.000	0.000	0.367

**Table 2:** Results of one-way ANOVA on the effects of Se concentration or treatment duration on glutathione reductase activity, reduced glutathione, and oxidized glutathione.

Glutathione redox cycle	Source		Se concentration (mg/L)			Treatment duration (d)			
			0	20	50		1	2	3
Glutathione reductase activity (nmol/min/mg protein)	Shoot	F	2392.345	1372.505	2371.150		954.975	1737.349	4399.808
		*p*	0.000	0.000	0.000		0.000	0.000	0.000
	Root	F	190.231	749.206	73.169		331.065	861.305	45.036
		*p*	0.000	0.000	0.000		0.000	0.000	0.000
Oxidized glutathione (nmol/g)	Shoot	F	942.484	455.995	987.889		604.229	157.521	1648.157
		*p*	0.000	0.000	0.000		0.000	0.000	0.000
	Root	F	308.100	348.800	234.402		133.697	692.820	149.867
		*p*	0.000	0.000	0.000		0.000	0.000	0.000
Reduced glutathione (nmol/g)	Shoot	F	131.699	202.031	310.746		830.788	3520.353	11.283
		*p*	0.000	0.000	0.000		0.000	0.000	0.000
	Root	F	101.841	47.722	73.791		1490.478	100.563	0.496
		*p*	0.000	0.000	0.000		0.000	0.000	0.773
GSH/GSSG	Shoot	F	126.232	207.044	296.315		1329.727	582.941	28.782
		*p*	0.000	0.000	0.000		0.000	0.000	0.000
	Root	F	108.822	52.854	63.779		1327.830	98.668	1.847
		*p*	0.000	0.000	0.000		0.000	0.000	0.178

**Table 3:** Glutathione reductase activity in the shoots and roots of *F. sinensis* with and without *Epichloë* endophyte under varying Na_2_SeO_3_ concentrations and treatment durations.

Se concentration (mg/L)	Plant	Glutathione reductase activity in shoot (nmol/min/mg protein)			Glutathione reductase activity in root (nmol/min/mg protein)		
		1 d	2 d	3 d	1 d	2 d	3d
0	EI	325.41 ± 5.99^Fe^	467.68 ± 5.29^Cb^	850.69 ± 5.99^Aa^	221.55 ± 6.19^Be^	485.31 ± 6.84^Bb^	522.60 ± 2.52^BCa^
	EF	452.77 ± 10.60^Ccd^	456.96 ± 1.00^CDbc^	443.19 ± 5.51^Dd^	146.51 ± 43.32^Cf^	321.84 ± 10.36^Dd^	410.93 ± 3.92^Dc^
20	EI	703.95 ± 12.38^Ab^	766.36 ± 5.36^Aa^	342.37 ± 2.08^Ee^	596.41 ± 12.68^Aa^	572.07 ± 5.92^Ab^	332.89 ± 9.14^Ed^
	EF	671.79 ± 6.19^Bc^	514.56 ± 10.72^Bd^	778.99 ± 6.19^Ba^	564.59 ± 6.10^Ab^	314.55 ± 1.53^De^	490.45 ± 7.51^Cc^
50	EI	368.05 ± 12.38^Dd^	455.72 ± 5.36^Dc^	848.88 ± 7.21^Aa^	579.12 ± 10.73^Aa^	412.75 ± 6.81^Cb^	592.75 ± 4.84^Aa^
	EF	350.19 ± 6.19^Ee^	316.93 ± 5.03^Ef^	638.63 ± 5.53^Cb^	564.59 ± 6.19^Aa^	283.53 ± 5.44^E^c	550.29 ± 59.04^ABa^

EI and EF represent *F. sinensis* infected with and without *Epichloë sinensis*, respectively. Means followed by different uppercase letters differ statistically within a given treatment time (*p* < 0.05). Means followed by different lowercase letters differ statistically at the same Se concentration (*p* < 0.05).

As time progressed following treatment, GR activity in the control and 50 mg/L Se-treated EI shoots, as well as in the control roots, exhibited significant increases throughout the experiment (**Table 3**, *p* < 0.05). In contrast, the GR activity in Se-treated EF shoots was markedly higher on the first day compared with the second day, but lower than on the third day (*p* < 0.05). The GR activities of the 50 mg/L Se-treated EI and EF roots were significantly greater on the first and third days than on the second day (*p* < 0.05). For the 20 mg/L Se-treated EI shoots, GR activity was significantly higher on the second day than on the first and third days, whereas the 20 mg/L Se-treated EF roots showed significantly lower activity on the second day compared with the first and third days (*p* < 0.05).

### 3.2 Effects on GSSG concentration

Except for the *Epichloë* endophyte’s effect on root GSSG, highly significant effects (*p* < 0.01, **Table 1**) of the *Epichloë* endophyte, Se concentration, and treatment duration were observed for GSSG in the shoots and roots of *F. sinensis*. Additionally, interactions between the *Epichloë* endophyte, Se concentration, and treatment duration were highly significant for GSSG in both shoots and roots (*p* < 0.01). On day 1, the EF shoot treated with 50 mg/L Se exhibited the highest level of GSSG (**Table 4**), whereas the EI root under 20 mg/L Se accumulated greater amounts of GSSG. Furthermore, the endophyte increased root GSSG under control conditions, as well as shoot and root GSSG at 20 mg/L Se. On day 2, the GSSG level in the EI shoot with 20 mg/L Se was significantly higher than in other treatments, and the EF root with 50 mg/L Se had the highest GSSG level among all treatments. At 20 mg/L Se, the endophyte enhanced GSSG in both shoots and roots. On day 3, the endophyte further increased the GSSG level in shoots and roots under both control and 20 mg/L Se treatments. The GSSG levels in EF shoots and roots treated with 50 mg/L Se were 42.67%–88.56% and 2.44%–18.01% higher than those in other treatments, respectively.

**Table 4:** Oxidized glutathione in the shoots and roots of *F. sinensis* with and without *Epichloë* endophyte under varying Na_2_SeO_3_ concentrations and treatment durations.

Se concentration (mg/L)	Plant	Oxidized glutathione in shoot (nmol/g)			Oxidized glutathione in root (nmol/g)		
		1 d	2 d	3 d	1 d	2 d	3 d
0	EI	616.36 ± 3.62^Eb^	564.43 ± 7.54^Dc^	744.38 ± 4.18^Ba^	511.29 ± 30.07^Ba^	455.73 ± 5.53^Dc^	452.11 ± 2.09^Ccd^
	EF	749.21 ± 4.18^Ca^	616.36 ± 3.62^Bb^	563.22 ± 4.18^Ec^	460.56 ± 40.60^Db^	450.90 ± 2.09^Dd^	429.16 ± 2.09^Ee^
20	EI	877.23 ± 13.06^Ba^	659.84 ± 9.59^Ac^	687.62 ± 7.54^Cb^	564.43 ± 2.09^Aa^	483.51 ± 5.53^Cc^	450.90 ± 4.18^Cd^
	EF	551.14 ± 7.25^Fe^	616.36 ± 7.25^Bd^	606.70 ± 9.12^Dd^	427.95 ± 6.28^Ee^	426.75 ± 4.18^Ee^	494.38 ± 5.53^Bb^
50	EI	683.99 ± 16.34^Db^	508.87 ± 2.09^Ee^	617.57 ± 4.18^Dc^	495.59 ± 10.46^Cc^	529.40 ± 3.62^Bb^	437.62 ± 2.09^Dd^
	EF	1,084.96 ± 24.66^Aa^	580.13 ± 9.59^Cd^	1,062.01 ± 13.06^Aa^	494.38 ± 11.65^Cc^	653.80 ± 9.12^Aa^	506.46 ± 7.54^Ac^

EI and EF represent *F. sinensis* infected with and without *Epichloë sinensis*, respectively. Means followed by different uppercase letters differ statistically within a given treatment time (*p* < 0.05). Means followed by different lowercase letters differ statistically at the same Se concentration (*p* < 0.05).

In relation to the time series, the GSSG levels in the control EF shoots and roots, as well as in the EI roots treated with 20 mg/L Se and without Se, exhibited significant increases. The levels of GSSG in the EI shoots with Se on day 3 were considerably higher than those on the second day, yet lower than those on the first day (*p* < 0.05). For the EF shoots treated with 50 mg/L Se, the GSSG levels on the second day were significantly lower than those on both the first and third days (*p* < 0.05). Conversely, the GSSG levels in the EF roots treated with 50 mg/L Se significantly increased (*p* < 0.05) on day 2 compared with day 1 and day 3. In the case of EF shoots treated with 20 mg/L Se, the GSSG levels significantly decreased (*p* < 0.05) on the first day compared with the second and third days, whereas the GSSG levels in the EF roots treated with 20 mg/L Se showed a sharp increase. Additionally, the EI roots treated with 50 mg/L Se exhibited lower GSSG levels on the first day compared with the second day, but higher levels than on the third day.

### 3.3 Effects on GSH concentration

Except for the GSH levels in the roots associated with the *Epichloë* endophyte, highly significant effects (*p* < 0.01, **Table 1**) of the *Epichloë* endophyte, Se concentration, and treatment duration were observed on the GSH levels in the shoots and roots of *F. sinensis*. Additionally, interactions were noted between the *Epichloë* endophyte and Se concentration regarding shoot GSH (*p* < 0.001), as well as between the *Epichloë* endophyte or Se concentration and treatment duration for root GSH. On the first day, the endophyte increased GSH levels in the shoots but decreased them in the roots (**Table 5**). Furthermore, the control EI shoots exhibited the highest levels of GSH, whereas the EF roots treated with 20 mg/L Se accumulated the most GSH among all treatments. On the second day, the endophyte enhanced the GSH levels in the shoots under both control and Se conditions, as well as in the roots treated with 20 mg/L Se. Moreover, GSH levels were higher in the EI shoots and roots treated with 20 mg/L Se compared with other treatments. On the 3rd day, the endophyte further increased the GSH levels in the shoots treated with 20 mg/L Se, which were the highest among all treatments.

**Table 5:** Reduced glutathione in the shoots and roots of *F. sinensis* with and without *Epichloë* endophyte under varying Na_2_SeO_3_ concentrations and treatment durations.

Se concentration (mg/L)	Plant	Reduced glutathione in shoot (nmol/g)			Reduced glutathione in root (nmol/g)		
		1 d	2 d	3 d	1 d	2 d	3 d
0	EI	1,661.88 ± 27.49^Ab^	943.22 ± 16.35^Bd^	2,951.50 ± 176.58^Ba^	814.01 ± 17.65^Cbc^	510.74 ± 25.67^Cc^	2,779.07 ± 323.88^Aa^
	EF	1281.97 ± 4.18^Bc^	903.05 ± 3.62^Cd^	2,756.19 ± 280.18^Ba^	1,017.67 ± 10.19^Bb^	521.45 ± 2.09^Cc^	2,731.43 ± 310.57^Aa^
20	EI	759.83 ± 21.59^Cd^	1,371.34 ± 9.59^Ac^	3,631.80 ± 238.12^Aa^	443.22 ± 15.87^Ed^	3,277.08 ± 303.83^Aa^	2,586.16 ± 324.29^Ab^
	EF	568.27 ± 16.86^Dd^	585.41 ± 7.25^Ed^	2,718.60 ± 298.46^BCb^	1,473.81 ± 14.10^Ac^	2,763.25 ± 330.00^Bb^	2,707.39 ± 328.42^Ab^
50	EI	394.24 ± 16.34^Ede^	604.66 ± 2.09^Dc^	2,595.96 ± 194.16^BCa^	559.12 ± 20.65^Dc^	3,019.42 ± 322.31^ABa^	2,511.21 ± 312.52^Ab^
	EF	322.69 ± 65.95^Fe^	533.40 ± 9.59^Fcd^	2,327.99 ± 142.32^Cb^	819.15 ± 18.02^Cc^	3,053.85 ± 16.33^ABa^	2,460.01 ± 305.36^Ab^

EI and EF represent *F. sinensis* infected with and without *Epichloë sinensis*, respectively. Means followed by different uppercase letters differ statistically within a given treatment time (*p* < 0.05). Means followed by different lowercase letters differ statistically at the same Se concentration (*p* < 0.05).

There was a time-dependent effect on GSH levels in both shoots and roots. The GSH concentrations in EI shoots treated with 20 and 50 mg/L Se, as well as in EF shoots treated with 50 mg/L Se, exhibited significant increases as the duration of treatment extended. Notably, EI roots treated with 20 and 50 mg/L Se, along with EF roots treated with 50 mg/L Se, displayed GSH levels on the third day that were lower than those on the second day but higher than those on the first day. Furthermore, the GSH levels in EF shoots supplied with 20 mg/L Se were greater on the third day than on both the first and second days, whereas EF roots under 20 mg/L Se exhibited higher GSH levels on the first day compared with the second and third days.

### 3.4 Effects on the GSH/GSSG ratio

Highly significant effects (*p* < 0.01) of the *Epichloë* endophyte, Se concentration, and treatment duration were observed for the GSH/GSSG ratio in the shoots of *F. sinensis* (**Table 1**). Additionally, interactions were noted between Se concentration and treatment duration, as well as among the three factors affecting the GSH/GSSG ratio in the shoots. Highly significant effects (*p* < 0.01) of Se concentration and treatment duration were also detected for the GSH/GSSG ratio in the roots. Notably, there were significant interactions between the *Epichloë* endophyte and treatment duration, as well as between Se concentration and treatment duration, regarding root GSH/GSSG ratios. After 1 day of Se treatment, the presence of the endophyte increased the GSH/GSSG ratios in the shoots under both control and 50 mg/L Se conditions (**Table 6**). Furthermore, the EI shoots without Se exhibited the highest GSH/GSSG ratios among all treatments. In contrast, the *Epichloë* endophyte reduced the GSH/GSSG ratios in the roots, with the EF treatment at 20 mg/L Se maintaining significantly higher ratios than those of other root samples. After 2 days of Se supplementation, the *Epichloë* endophyte significantly elevated the GSH/GSSG ratios in both shoots and roots, except for the roots under control conditions. Moreover, the GSH/GSSG ratios of EI shoots and roots supplied with 20 mg/L Se were significantly higher than those of other plants. After 3 days of Se supplementation, the *Epichloë* endophyte significantly increased the GSH/GSSG ratios in the shoots under Se addition, with the EI shoot at 20 mg/L Se exhibiting the highest ratios.

**Table 6:** Reduced glutathione/oxidized glutathione (GSH/GSSG) in the shoots and roots of *F. sinensis* with and without *Epichloë* endophyte under varying Na_2_SeO_3_ concentrations and treatment durations.

Se concentration (mg/L)	Plant	GSH/GSSG in shoot			GSH/GSSG in root		
		1 day	2 days	3 days	1 day	2 days	3 days
0	EI	2.70 ± 0.03^Ac^	1.67 ± 0.05^Bd^	3.97 ± 0.25^Cb^	1.59 ± 0.03^Cbc^	1.12 ± 0.07^Dc^	6.15 ± 0.72^Aa^
	EF	1.71 ± 0.02^Cd^	1.47 ± 0.01^Cd^	4.89 ± 0.47^ABa^	2.21 ± 0.02^Bb^	1.16 ± 0.01^Dc^	6.36 ± 0.70^Aa^
20	EI	0.87 ± 0.04^Dd^	2.08 ± 0.04^Ac^	5.28 ± 0.33^Aa^	0.79 ± 0.03^Ee^	6.77 ± 0.55^Aa^	5.74 ± 0.73^Abc^
	EF	1.93 ± 0.04^Bd^	0.95 ± 0.02^Ed^	4.48 ± 0.05^BCb^	3.44 ± 0.02^Ad^	6.47 ± 0.73^ABab^	5.47 ± 0.63^Ac^
50	EI	0.58 ± 0.04^Ee^	1.19 ± 0.01^Dc^	4.20 ± 0.32^Ca^	1.13 ± 0.06^Dc^	5.70 ± 0.60^Ba^	5.74 ± 0.72^Aa^
	EF	0.30 ± 0.07^Ff^	0.92 ± 0.03^Ed^	2.19 ± 0.15^Db^	1.66 ± 0.07^Cc^	4.67 ± 0.07^Cb^	4.85 ± 0.54^Ab^

EI and EF represent *F. sinensis* infected with and without *Epichloë sinensis*, respectively. Means followed by different uppercase letters differ statistically within a given treatment time (*p* < 0.05). Means followed by different lowercase letters differ statistically at the same Se concentration (*p* < 0.05).

A significant time effect on GSH/GSSG ratios in shoot and root were also observed. The GSH/GSSG ratios in EI shoots with Se and EF shoots with 50 mg/L Se sharply increased as the time passed, EF shoots with 0 and 20 mg/L Se and EI roots without Se for the GSH/GSSG ratios were higher on the third than on the first and second days, and the GSH/GSSG ratios EI and EF roots with 50 mg/L Se on the first day were lower than on the second and third days. In addition, the GSH/GSSG ratios of EI and EF roots with 20 mg/L Se were higher on the third day than on the first day, but lower than on the second day.

### 3.5 Effects of Se on the expression of glutathione-metabolizing genes in shoots

A total of 26,754 unigenes were identified from 108 samples, with a mean length 3,290.46 bp and an N50 length of 4,501 bp. A total of 6,406 unigenes were annotated within eight different public databases, including COG, KOG, eggnog, and Nr databases (**Supplementary Figure 1**). A total of 6, 25, 947, and 267 unigenes were unique to GO, KEGG, Pfam, and Swiss-Prot, respectively. The results indicated that expression of all unigenes differed between EI and EF plants in terms of Se treatments and treatment duration (**Supplementary Table 1**).

KEGG pathway results indicated that these DEGs are involved in glutathione metabolism such as for GSH synthesis (*GCLC*, *GS*), degradation (*GGT*, *GGCT*, EC:3.4.13.-, *5-OPase*), recycling (*GR*, *GPX*), and the metabolism of antioxidants (*GST*, *APX*) (**Supplementary Figure 2**). The differential gene expression patterns of EI and EF shoots under the normal and Se supplementation are presented in **Supplementary Table 2**. The comparison between EFCK1 and EF201 groups revealed the lowest number of DEGs, with three upregulated and two downregulated genes. Conversely, the EFCK3 *vs*. EF503 group exhibited the highest number of DEGs, with 17 upregulated and 20 downregulated genes. Compared with the control EI on the first day, 27 and 19 DEGs in EI shoots were upregulated under the 20 and 50 mg/L Se conditions, respectively, whereas only one DEG and one DEG were downregulated, respectively. Furthermore, 10 and 15 DEGs were upregulated whereas 16 and seven DEGs were downregulated for EICK2 *vs*. EI202 and EICK2 *vs*. EI502, respectively. Additionally, only nine upregulated differential genes were observed in EI shoots between the 20 and 50 mg/L Se-treated and control groups on the third day. In EF shoots, 32 significantly different genes were found between the 50 mg/L Se-treated and control groups, comprising 31 upregulated and one downregulated genes. Additionally, 6 and 12 DEGs were respectively upregulated under the 20 and 50 mg/L Se supplementation, whereas 18 and nine DEGs were downregulated compared with the control on the second day, respectively. In the comparison between EFCK3 and EF203 groups, 22 DEGs were identified, with 1 upregulated gene and 21 downregulated genes.

**Figure 1:**
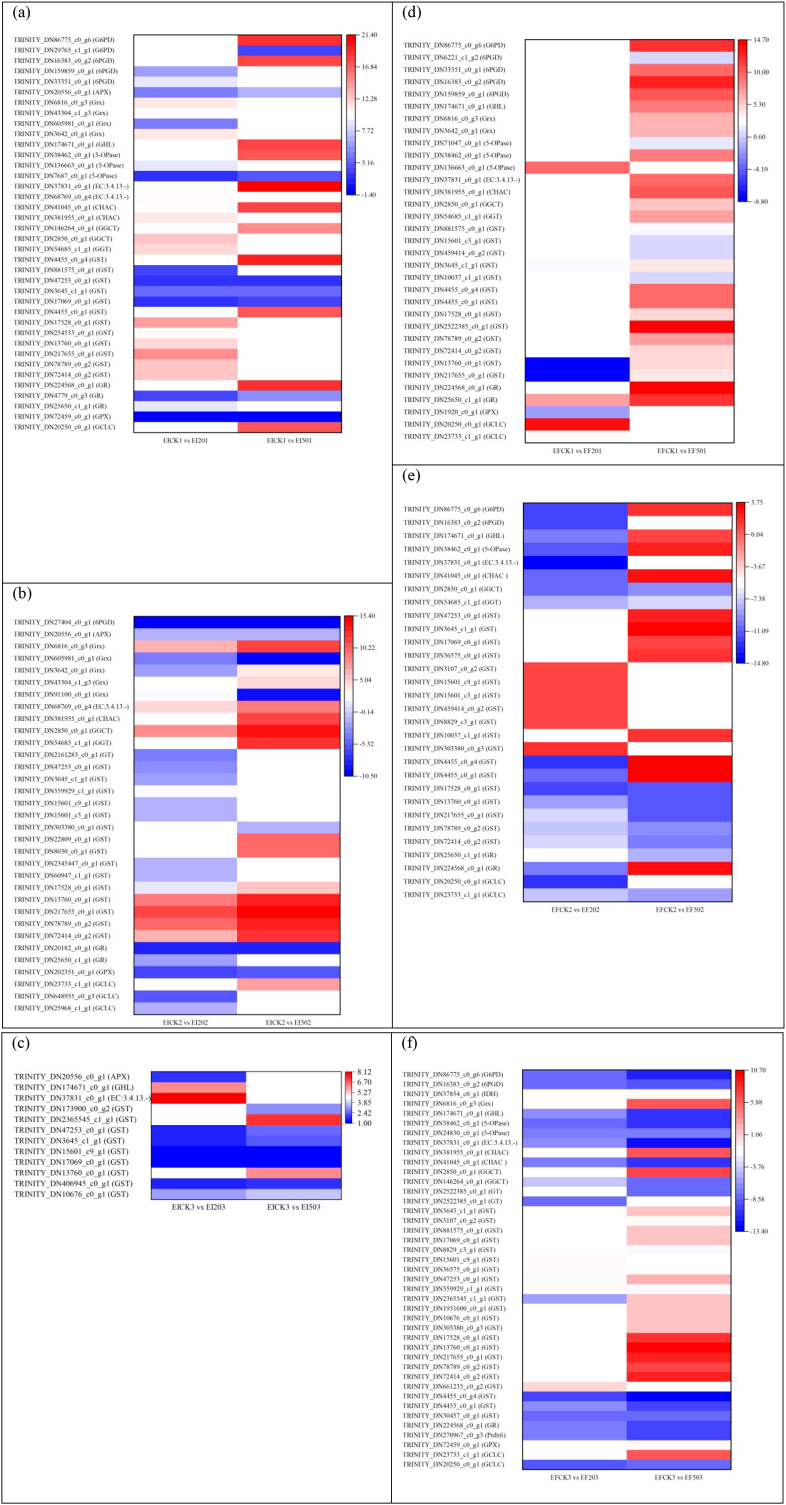
Differentially expressed genes associated with glutathione metabolism in shoot comparisons without Se (CK) *vs*. 20 or 50 mg/L Se at three different time points. **(a)** EICK1 *vs*. EI201 and EICK1 *vs*. EI501, **(b)** EICK2 *vs*. EI202 and EICK2 *vs*. EI502, **(c)** EICK3 *vs*. EI203 and EICK3 *vs*. EI503, **(d)** EFCK1 *vs*. EF201 and EFCK1 *vs*. EF501, **(e)** EFCK2 *vs*. EF202 and EFCK2 *vs*. EF502, and **(f)** EFCK3 *vs*. EF203 and EFCK3 *vs*. EF503. Each column represents a different sample characterized by endophyte status, Se concentration, and treatment duration. Red and blue represent upregulated and downregulated expression, respectively, whereas white indicates no expression. EICK1 *vs*. EI201 represents the comparison of DEGs in the shoot of endophyte-infected *F. sinensis* supplied without and with 20 mg/L Na_2_SeO_3_ on day 1; EICK1 *vs*. EI501 represents the comparison of DEGs in the shoot of endophyte-infected *F. sinensis* supplied without and with 50 mg/L Na_2_SeO_3_ on day 1; EICK2 *vs*. EI202 represents the comparison of DEGs in the shoot of endophyte-infected *F. sinensis* supplied without and with 20 mg/L Na_2_SeO_3_ on day 2; EICK2 *vs*. EI502 represents the comparison of DEGs in the shoot of endophyte-infected *F. sinensis* supplied without and with 50 mg/L Na_2_SeO_3_ on day 2; EICK3 *vs*. EI203 represents the comparison of DEGs in the shoot of endophyte-infected *F. sinensis* supplied without and with 20 mg/L Na_2_SeO_3_ on day 3; EICK3 *vs*. EI503 represents the comparison of DEGs in the shoot of endophyte-infected *F. sinensis* supplied without and with 50 mg/L Na_2_SeO_3_ on day 3; EFCK1 *vs*. EF201 represents the comparison of DEGs in the shoot of endophyte-free *F. sinensis* supplied without and with 20 mg/L Na_2_SeO_3_ on day 1; EFCK1 *vs*. EF501 represents the comparison of DEGs in the shoot of endophyte-free *F. sinensis* supplied without and with 50 mg/L Na_2_SeO_3_ on day 1; and REICK1 *vs*. REI201 represents the comparison of DEGs in the root of endophyte-infected *F. sinensis* supplied without and with 20 mg/L Na_2_SeO_3_ on day 1. And so on.

As shown in **Figures 1a**, there were eight overlapping DEGs regulated in the shoots between the comparisons EICK1 *vs*. EI201 and EICK1 *vs*. EI501. The expression levels of TRINITY_DN25968_c1_g1 (glutamate-cysteine ligase catalytic subunit, *GCLC*), TRINITY_DN4779_c0_g3 (glutathione reductase, *GR*), TRINITY_DN17069_c0_g1, TRINITY_DN3645_c1_g1, TRINITY_DN47253_c0_g1 (glutathione S-transferase, *GST*), TRINITY_DN7687_c0_g1 (5-oxoprolinase, *5-OPase*), and TRINITY_DN20556_c0_g1 (L-ascorbate peroxidase, *APX*) were upregulated by 2.99–5.32-, 2.28–5.37-, 1.59–1.94-, 3.45–3.38-, 1.36–1.14-, 1.46–2.41-, 4.19–6.30-fold (log2), respectively, under Se supplementations, but TRINITY_DN72459_c0_g1 encoding glutathione peroxidase (*GPX*) was downregulated by −1.36- or −1.37-fold. The EICK2 *vs*. EI202 and EICK2 *vs*. EI502 comparisons showed 14 common DEGs (**Figure 1b**), including *GPX* (TRINITY_DN202351_c0_g1), *GR* (TRINITY_DN20182_c0_g1), *APX* (TRINITY_DN20556_c0_g1), 6-phosphogluconate dehydrogenase (*6PGD*, TRINITY_DN27404_c0_g1), gamma-glutamylcyclotransferase (*GGCT*, TRINITY_DN2850_c0_g1), *GST* (TRINITY_DN72414_c0_g2, TRINITY_DN78789_c0_g2, TRINITY_DN217655_c0_g1, TRINITY_DN13760_c0_g1, TRINITY_DN17528_c0_g1), and glutaredoxin (*Grx*, TRINITY_DN605981_c0_g1, TRINITY_DN6816_c0_g3, TRINITY_DN91100_c0_g1, TRINITY_DN3642_c0_g1). The four genes encoding *GPX*, *GR*, *APX*, and *6PGD* showed the reductions of −6.43 to −6.00, −7.98 to −8.41, −1.69 to −1.54, and −10.26 to −9.83, respectively. Lower levels of expression for the four genes were observed in the presence of 20 mg/L Se than 50 mg/L Se. However, five genes encoding *GST* and one gene related to *GGCT* increased to 1.51–15.32- and 8.25–13.87-fold (log2). The six upregulated genes showed higher levels of gene expressions in EI shoot under 50 mg/L Se than under 20 mg/L Se. Additionally, two of four genes, which were involved in *Grx*, had the same variation trend under the two Se concentrations, but two other genes showed an inconsistent trend. On day 3, there were six shared genes, which encode glutathione S-transferase between the comparisons EICK3 *vs*. EI203 and EICK3 *vs*. EI503 (**Figure 1c**). The six genes increased to 1.01–3.62-fold (log2).

In total, 4 DEGs were regulated in both EFCK1 *vs*. EF201 and EFCK1 *vs*. EF501 comparisons, 15 DEGs in both EFCK2 *vs*. EF202 and EFCK2 *vs*. EF502 comparisons, and 18 DEGs in both EFCK3 *vs*. EF203 and EFCK3 *vs*. EF503 comparisons (**Figures 1d–f**). In both EFCK1 *vs*. EF201 and EFCK1 *vs*. EF501 comparisons (**Figure 1d**), TRINITY_DN25650_c1_g1 and TRINITY_DN3645_c1_g1 were respectively annotated as *GR* and *GST* and both were 7.38–12.08- and 2.59–4.38-fold upregulated, respectively, but for the other two genes related to *GST*, both were downregulated (−8.80 to −8.44) in EFCK1 *vs*. EF201 but upregulated (4.37–4.75-fold) in EFCK1 *vs*. EF501. In both EFCK2 *vs*. EF202 and EFCK2 *vs*. EF502 comparisons (**Figure 1e**), eight overlapping genes including *GCLC*, *GST* with five members, *GGT*, and *GGCT* were found to downregulate in both comparisons; seven other common genes *GR*, *GST*, *GST*, *CHAC*, *5-OPase*, *GHL*, and *G6PD* were significantly upregulated in the presence of 50 mg/L Se with increases of 1.17–3.62-fold and downregulated in the presence of 20 mg/L Se with reductions of −12.79- to −9.96-fold. In both EFCK3 *vs*. EF203 and EFCK3 *vs*. EF503 comparisons (**Figure 1f**), 16 of 18 overlap DEGs including *Prdx6*, *GST*, *GGCT*, Cys-Gly metallodipeptidase (EC:3.4.13.-), *5-OPase*, *IDH*, and *6PGD* showed reductions by −1.18- to −13.37-fold, whereas another two genes encoding *GST* differed in the response to 20 and 50 mg/L Se.

The time-course analysis revealed the dynamics of upregulated and downregulated genes in shoots, as presented in **Supplementary Table 2**. In comparison with the first day, EI shoots exhibited differential expression of 21 transcripts (19 up and two down) on the second day under standard conditions, which reduced to eight transcripts on the third day. In addition, the number of differentially expressed transcripts in EF shoot was 29 on the second day and elevated to 34 on the third day. Under the 20 mg/L Se treatment, a total of 24 DEGs in shoot were identified between the EI201 and EI202 groups, 39 DEGs between the EI201 and EI203 groups. Conversely, in the EF201 *vs*. EF202 group three upregulated and five downregulated DEGs were observed, whereas in the EF201 *vs*. EF203 group, five upregulated and four downregulated DEGs were found. Upon the application of 50 mg/L Na_2_SeO_3_, a total of 36 DEGs were identified between the EI501 and EI502 groups with 18 upregulated and 18 downregulated genes, 36 DEGs consisting of 9 downregulated and 17 upregulated ones between the EI501 and EI503 groups, and 2 upregulated and 20 downregulated DEGs between the EF501 and EF502 groups; notably, there were 16 upregulated and 25 downregulated DEGs in the EF501 *vs*. EF503 comparisons. In plant shoots, we identified 302 upregulated and 250 downregulated DEGs.

**Figure 2:**
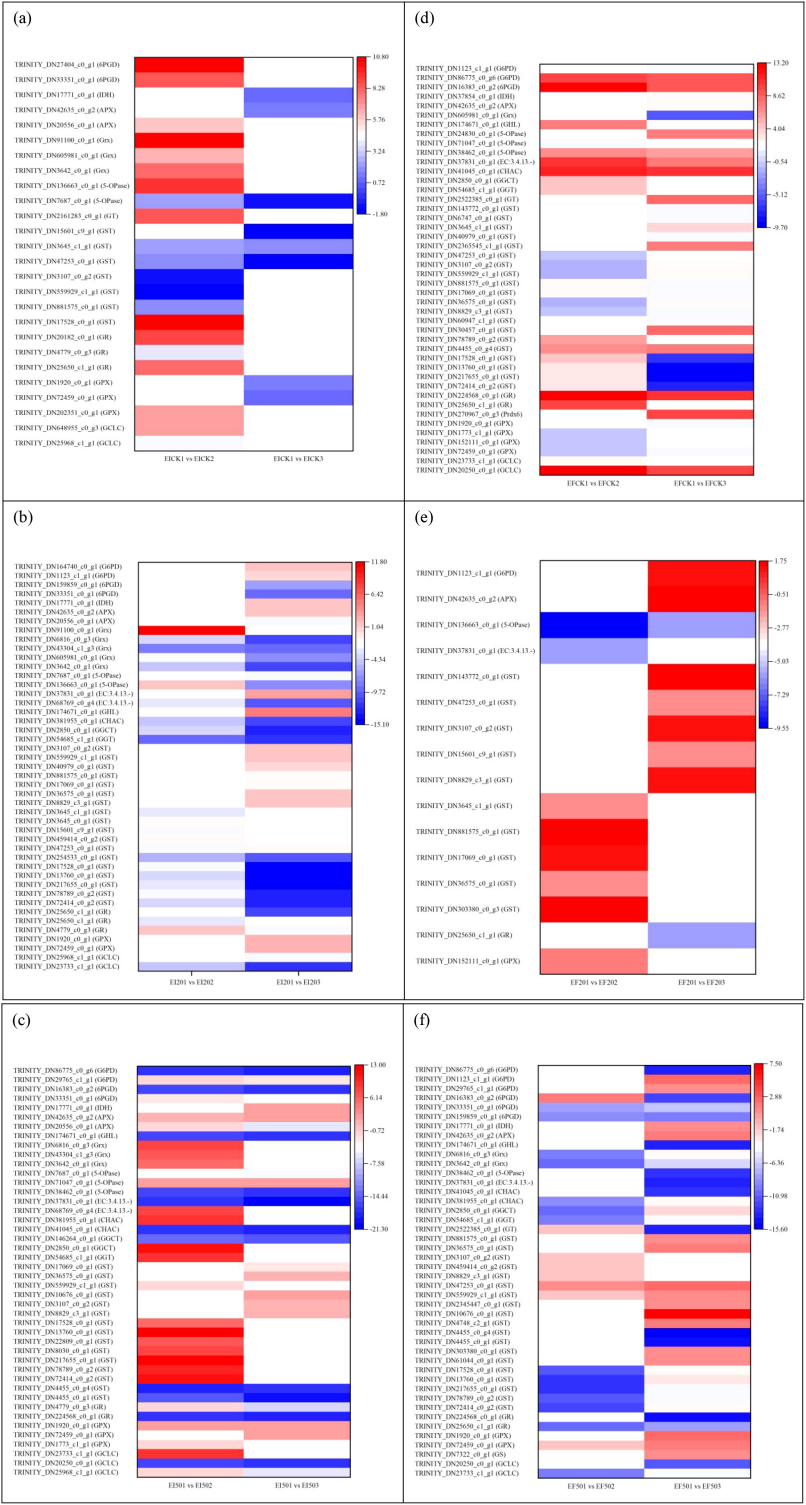
Differentially expressed genes associated with glutathione metabolism in shoots at 1 day *vs*. 2 days or 3 days. **(a)** EICK1 *vs*. EICK2 and EICK1 *vs*. EICK3, **(b)** EI201 *vs*. EI202 and EI201 *vs*. EI203, **(c)** EI501 *vs*. EI502 and EI501 *vs*. EI503, **(d)** EFCK1 *vs*. EFCK2 and EFCK1 *vs*. EFCK3, **(e)** EF201 *vs*. EF202 and EF201 *vs*. EF203, and **(f)** EF501 *vs*. EF502 and EF501 *vs*. EF503. Red and blue represent upregulated and downregulated expression, respectively, whereas white indicates no expression. Abbreviations of the samples are the same as in **Figure 1**.

Notably, only three DEGs were observed in the EICK1 *vs*. EICK2 and EICK1 *vs*. EICK3 groups (**Figure 2a**), the genes were upregulated by 1.65–2.35-fold in EICK1 *vs*. EICK2, and only *GST* was upregulated by 1.75-fold in the EICK1 *vs*. EICK3 group. Out of 18 common DEGs in the EI201 *vs*. EI202 and EI201 *vs*. EI203 groups, 16 showed a reduction of −12.95- to −1.74-fold (**Figure 2b**); these genes, except for TRINITY_DN47253_c0_g1 encoding *GST*, had greater expressions in the EI201 *vs*. EI202 group. Both the EI501 *vs*. EI502 and EI501 *vs*. EI503 groups shared 18 DEGs with the same trend (**Figure 2c**); three upregulated genes related to *Grx*, *5-OPase*, and *APX* exhibited a higher expression in the EI501 *vs*. EI503 groups, but 15 downregulated genes in the EI501 *vs*. EI502 group showed higher expression levels, which were assigned to *GR*, *GST*, *G6PD*, *6PGD*, and EC:3.4.13.-.

For EFCK1 *vs*. EFCK2 and EFCK1 *vs*. EFCK3 (**Figure 2d**), 19 DEGs overlapped, and 10 upregulated ones except for TRINITY_DN4455_c0_g4 had higher expression levels on day 2, whereas nine DEGs were reversed in trend in both groups. However, only one DEG involved in *5-OPase* in both the EF201 *vs*. EF202 and EF201 *vs*. EF203 groups was downregulated from −9.50 to −6.16 (log2 FC) (**Figure 2e**). Additionally, the EF501 *vs*. EF502 and EF501 *vs*. EF503 groups shared 19 DEGs, and most of the 16 downregulated DEGs had lower expression levels in the EF501 *vs*. EF502 group. TRINITY_DN72459_c0_g1 and TRINITY_DN559929_c1_g1 encoding *GPX* and *GST* were upregulated under 50 mg/L Se on the third day compared with the first day (**Figure 2f**), whereas TRINITY_DN16383_c0_g2 related to *6PGD* was upregulated on the second day. These findings thus revealed that treatment time can significantly affect the glutathione-metabolizing gene in EF shoots in the presentation of 50 mg/L Se than in the presentation of 0 and 20 mg/L Se.

### 3.6 Effects of *Epichloë* endophyte the expression of glutathione-metabolizing genes in shoots

**Figure 3:**
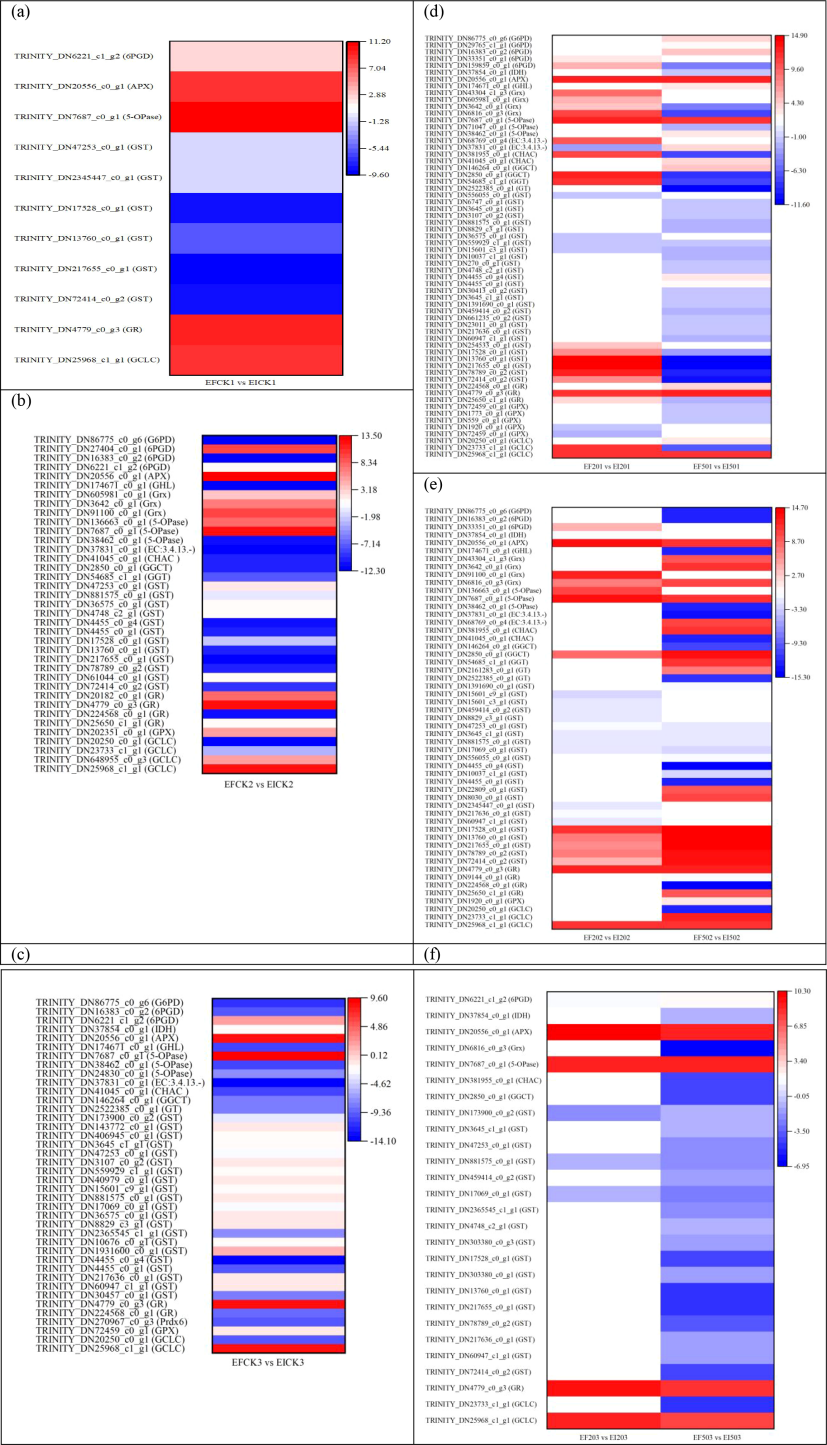
Differentially expressed genes associated with glutathione metabolism in shoot EF *vs*. EI groups. **(a)** EFCK1 *vs*. EICK1, **(b)** EFCK2 *vs*. EICK2, **(c)** EFCK3 *vs*. EICK3, **(d)** EF201 *vs*. EI201 and EF501 *vs*. EI501, **(e)** EF202 *vs*. EI202 and EF502 *vs*. EI502, and **(f)** EF203 *vs*. EI203 and EF503 *vs*. EI503. Red and blue represent upregulated and downregulated expression, respectively, whereas white indicates no expression. Abbreviations of the samples are the same as in **Figure 1**.

In this experiment, the transcripts of EI shoots were compared with EF shoots (**Supplementary Table 2**, **Figure 3**). We observed six and five downregulated and upregulated genes in the EFCK1 *vs*. EICK1 groups (**Figure 3a**), respectively. A total of 19 and 18 genes downregulated as well as upregulated in the EFCK2 *vs*. EICK2 groups (**Figure 3b**). Only six genes in all 40 DEGs were upregulated in the EFCK3 *vs*. EICK3 groups (**Figure 3c**). The amount of DEGs under control conditions continued to increase as time passed.

A total of 22 genes were upregulated, whereas seven genes were downregulated in the EF201 *vs*. EI201 groups. In the EF202 *vs*. EI202 groups, 14 genes were upregulated whereas 12 genes were downregulated. However, only five genes in all eight DEGs were upregulated in the EF203 *vs*. EI203 groups.

Among the 53 DEGs between the EF shoot and EI shoot groups with 50 mg/L Se treatment for 1 day, 16 were up-expressed and 37 were down-expressed. In addition, there were 42 DEGs detected for 2 days of treatment, including 22 up-expressed and 20 down-expressed unigenes. Under 50 mg/L Se exposure for 3 days, only five genes in all 27 DEGs were upregulated in the EF503 *vs*. EI503 groups. Expression levels of genes in the EF *vs*. EI shoot with 50 mg/L Se were more active for 1–2 days than those for 3 days. The amount of DEGs for Se treatment decreased as the time series.

In both EF201 *vs*. EI201 and EF501 *vs*. EI501, 20 DEGs were commonly regulated (**Figure 3d**). Six of the 20 shared consistency variations were *GCLC*, *GR*, *5-OPase*, *APX*, and two *GST*. Among the 20 overlapping genes, we found that most genes were upregulated and more highly expressed in the EF201 *vs*. EI201 group, including GSH biosynthesis and degrading genes (*GCLC*, *GR*, *GST*, *GGT*, *GGCT*, *Grx*, *5-OPase*, and *6PGD*). On the other hand, eight DEGs were shared in the three EFCK1 *vs*. EICK1, EF201 *vs*. EI201, and EF501 *vs*. EI501 comparisons (**Figures 3a, d**), showing the same trend between EFCK1 *vs*. EICK1 and EF501 *vs*. EI501 groups.

A total of 15 DEGs were identified for the overlapping genes and had the same trend of expression in EF202 *vs*. EI202 and EF502 *vs*. EI502 (**Figure 3e**), whereas 11 genes were upregulated, and four other genes encoding *GST* were downregulated. Additionally, most genes were more highly expressed in the EF202 *vs*. EI202 group. Interestingly, 11 DEGs were shared among EFCK2 *vs*. EICK2, EF202 *vs*. EI202, and EF502 *vs*. EI502 (**Figures 3b, e**), with a difference in the EFCK2 *vs*. EICK2 group.

In the EFCK3 *vs*. EICK3, EF203 *vs*. EI203, and EF503 *vs*. EI503 comparisons (**Figures 3c, f**), eight DEGs were shared and showed the same trend. the five genes respectively involved in *6PGD*, *GCLC*, *GR*, *5-OPase*, and *APX* were upregulated from 1.49 to 10.28 (log2 FC). Three downregulated genes could be assigned to *GST* with reduction of −3.49 to −1.05 fold (log2).

### 3.7 Effects of Se on the expression of glutathione-metabolizing genes in roots

In the REICK1 *vs*. REI201 group, there were 57 DEGs, with 36 upregulated and 21 downregulated (**Supplementary Table 2**). We observed 28 DEGs in the REICK1 *vs*. REI501 group, with only 13 DEGs increasing and 15 decreasing. A total of 47 DEGs consisted of 21 upregulated and 26 downregulated genes in the REFCK1 *vs*. REF201 group. Furthermore, there were 58 DEGs with 31 upregulated and 27 downregulated in the REFCK1 *vs*. REF501 group.

In the REICK2 *vs*. REI202 group, 40 DEGs were obtained, of which 33 were upregulated and seven were downregulated. Additionally, in the REICK2 *vs*. REI502 group, 45 DEGs were found, comprising 34 upregulated and 11 downregulated genes. By contrast, in the EF roots, 26 and 23 DEGs were respectively downregulated under the 20- and 50-mg/L Se supplementation, whereas 7 and 16 DEGs were upregulated compared with the control on the second day, respectively. On the third day, most DEGs in roots were significantly upregulated under Se supplementation. Compared with REICK3, 35 DEGs were identified in REI203 and 42 DEGs were identified in REI503. In comparison with REFCK3, 33 upregulated and 19 downregulated DEGs were identified in REF203, and 32 upregulated and 22 downregulated DEGs were identified in REF503.

**Figure 4:**
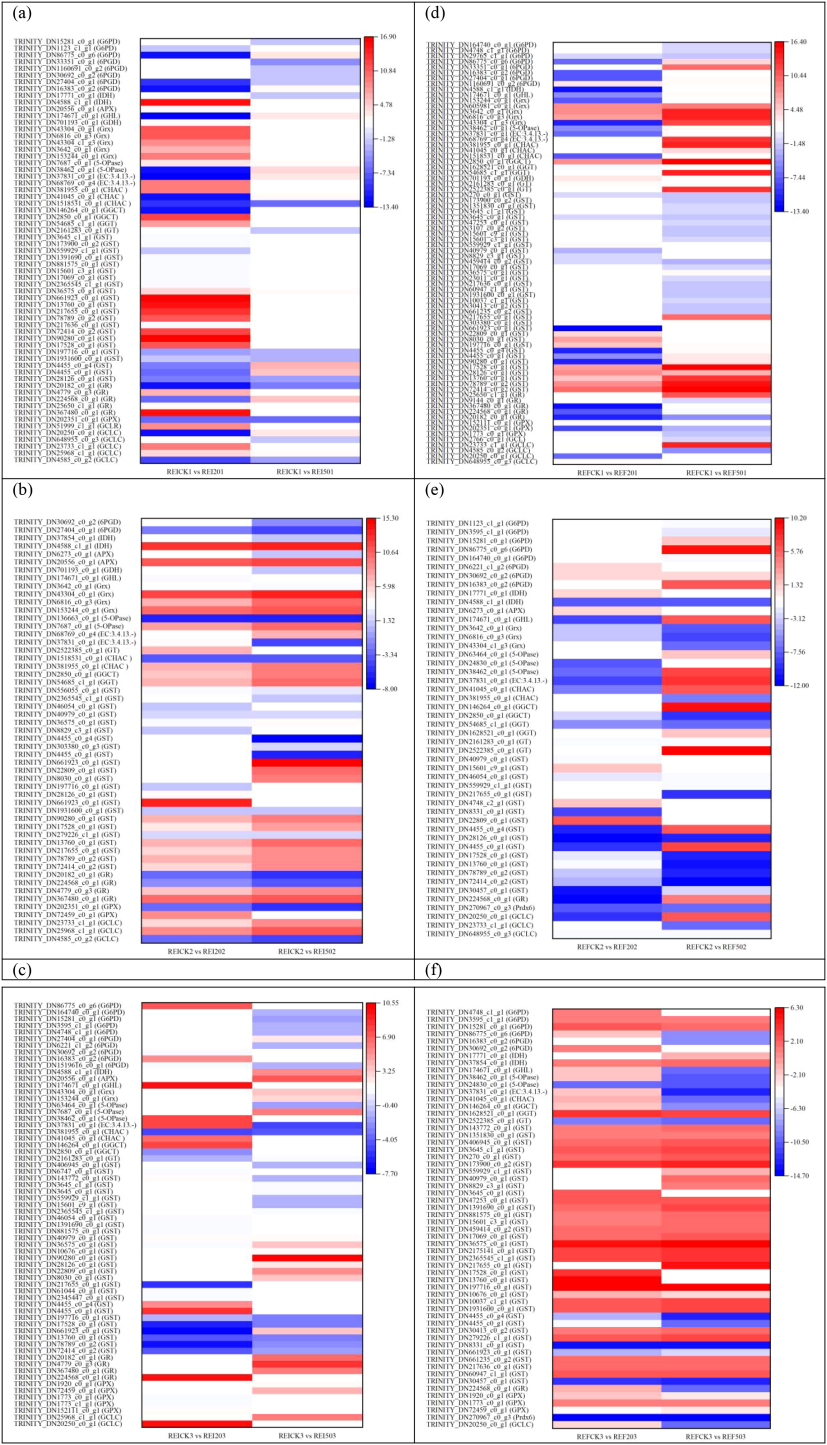
Differentially expressed genes associated with glutathione metabolism in roots: control *vs*. Se treatment. **(a)** REICK1 *vs*. REI201 and REICK1 *vs*. REI501, **(b)** REICK2 *vs*. REI202 and REICK2 *vs*. REI502, **(c)** REICK3 *vs*. REI203 and REICK3 *vs*. REI503, **(d)** REFCK1 *vs*. REF201 and REFCK1 *vs*. REF501, **(e)** REFCK2 *vs*. REF202 and REFCK2 *vs*. REF502, and **(f)** REFCK3 *vs*. REF203 and REFCK3 *vs*. REF503. Each column represents a different sample characterized by endophyte status, Se concentration, and treatment duration. Red and blue represent upregulated and downregulated expression, respectively, whereas white represents no expression. Abbreviations of the samples are the same as in **Figure 1**.

The REICK1 *vs*. REI201 and REICK1 *vs*. REI501 groups shared 21 DEGs (**Figure 4a**), 11 DEGs including three upregulated (*GCLC*, *GST*, and *5-OPase*) and eight downregulated showed the same trend, 10 other DEGs were reversed in both groups. All these genes had far greater expression levels under 50 mg/L Se than under 20 mg/L Se. There were 31 of 32 overlapped DEGs in both the REICK2 *vs*. REI202 and REICK2 *vs*. REI502 groups that showed the same trend encompassing 24 upregulated and seven downregulated ones (**Figure 4b**), in which most of the shared DEGs had a higher expression responding to 50 mg/L Se. Some pronounced differences were identified in the REICK3 *vs*. REI203 and REICK3 *vs*. REI503 groups (**Figure 4c**), where 13 overlapped and 22 were REICK3 *vs*. REI203 group specific, whereas 29 were REICK3 *vs*. REI503 group specific. Only two of the shared 13 DEGs (TRINITY_DN36575_c0_g1 and TRINITY_DN40979_c0_g1) were upregulated and six downregulated in both groups. However, the other five genes exhibited the opposite trend in both groups.

Both the REFCK1 *vs*. REF201 and REFCK1 *vs*. REF501 groups had 28 common DEGs (**Figure 4d**), consisting of 17 similar changes and 11 opposite changes. There were 11 of 17 DEGs that were upregulated 2.14–16.38-fold (log2 FC), but others were downregulated by −10.77- to −1.12-fold (log2). Additionally, 10 of the opposite 11 DEGs exhibited increases 1.17–11.90-fold (log2) in the REFCK1 *vs*. REF501 group. The REFCK2 *vs*. REF202 and REFCK2 *vs*. REF502 groups had 24 overlapped genes including 16 similar and 8 different changes (**Figure 4e**). Only TRINITY_DN30692_c0_g2 had an upregulated expression; the other 15 shared DEGs had downregulated expressions −8.24- to −1.14-fold (log2 FC). The eight different changes were upregulated 4.47–7.63-fold (log2) in the REFCK2 *vs*. REF502 group. In contrast, more pronounced similarities were identified in the REFCK3 *vs*. REF203 and REFCK3 *vs*. REF503 groups (**Figure 4f**), where 47 DEGs overlapped including 28 upregulated and 19 downregulated, but only five and six were specific for the REFCK3 *vs*. REF203 and REFCK3 *vs*. REF503 groups, respectively.

DEGs varied across different time intervals when roots were exposed to either common or Se conditions (**Supplementary Table 2**). A comparison with the day 1 control EI root revealed that in the REICK2 and REICK3 groups, 8 upregulated and 22 downregulated DEGs and 22 upregulated and 29 downregulated DEGs were identified, respectively. Under supplementation with 20 mg/L Na_2_SeO_3_, 58 and 66 DEGs were observed in the REI201 *vs*. REI202 and REI201 *vs*. REI203 groups, respectively. Similarly, in the REI501 *vs*. REI502 group, 25 upregulated and 17 downregulated were observed. Conversely, in the REI501 *vs*. REI503 group, 8 upregulated and 14 downregulated DEGs were identified, respectively.

While a total of 40 DEGs were significantly modulated in the REFCK1 *vs*. REFCK2 groups (**Supplementary Table 2**), 59 were found in the REFCK1 *vs*. REFCK3 groups. Among those, 26 were upregulated and 14 downregulated in the former, whereas 13 were upregulated and 46 were downregulated in the latter with a wider distribution of DEGs. The comparison revealed 26 upregulated and 15 downregulated genes in the REFCK1 *vs*. REFCK2 samples, and 13 upregulated and 46 downregulated genes in the REFCK1 *vs*. REFCK3 samples. We detected 15 DEGs that were upregulated among the 24 DEGs between REF201 and REF202 groups, and 28 DEGs that were downregulated among the 40 DEGs between REF201 and REF203 groups. There were 13 DEGs in the REF501 *vs*. REF502 group; the expression level of four DEGs rose, whereas nine DEGs reduced. In the REF501 *vs*. REF503 group, 21 DEGs were identified with 5 upregulated genes and 16 downregulated genes. For plant roots, we identified 508 upregulated and 490 downregulated DEGs.

**Figure 5:**
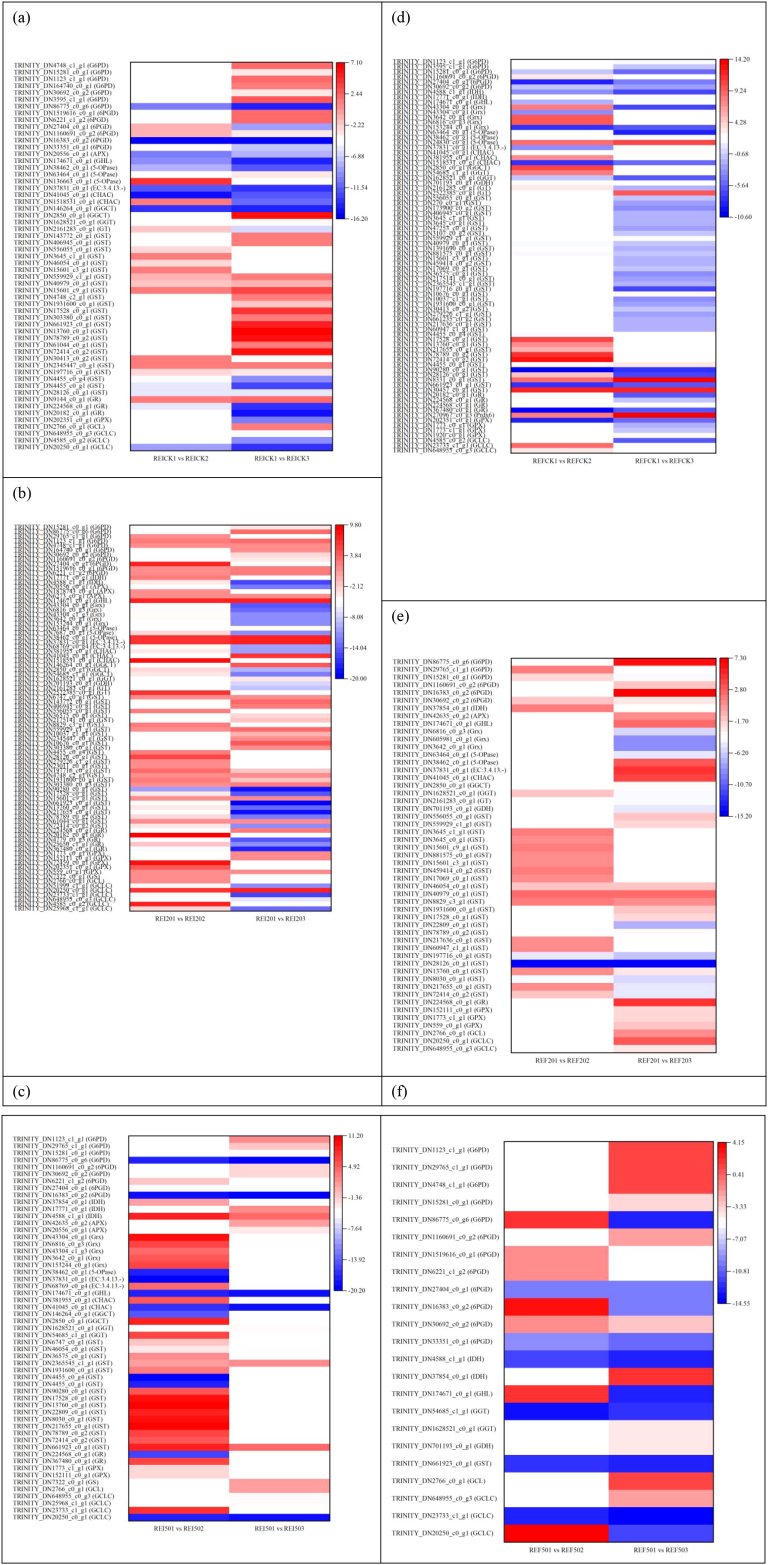
Differentially expressed genes associated with glutathione metabolism in roots at 1 day *vs*. 2 days or 3 days. **(a)** REICK1 *vs*. REICK2 and REICK1 *vs*. REICK3, **(b)** REI201 *vs*. REI202 and REI201 *vs*. REI203, **(c)** REI501 *vs*. REI502 and REI501 *vs*. REI503, **(d)** REFCK1 *vs*. REFCK2 and REFCK1 *vs*. REFCK3, **(e)** REF201 *vs*. REF202 and REF201 *vs*. REF203, and **(f)** REF501 *vs*. REF502 and REF501 *vs*. REF503. Red and blue represent upregulated and downregulated expression, respectively, whereas white represents no expression. Abbreviations of the samples are the same as in **Figure 1**.

The REICK1 *vs*. REICK2 and REICK1 *vs*. REICK3 groups had 24 common DEGs (**Figure 5a**), in which 22 had the same trend consisting of 3 upregulated (1.19–3.44-fold) and 19 downregulated (−16.18- to −1.50-fold). Most overlapped DEGs had greater expression levels in the REICK1 *vs*. REICK2 group. A total of 40 DEGs were common to both the REI201 *vs*. REI202 and REI201 *vs*. REI203 groups (**Figure 5b**), in which 38 DEGs had a similar variation and 2 DEGs showed the opposite trend. In addition, 16 upregulated DEGs except for TRINITY_DN61044_c0_g1 had far greater expression levels after a 3-day exposure than after a 2-day exposure, but the other 22 downregulated DEGs had lower expression levels after a 3-day exposure than after a 2-day exposure. However, only nine common DEGs were found between the REI501 *vs*. REI502 and REI501 *vs*. REI503 groups (**Figure 5c**), including three upregulated (1.56–8.44-fold) and six downregulated (−19.38- to −4.95-fold) genes. The expression levels of most genes were higher in the former than the later groups.

Both REFCK1 *vs*. REFCK2 and REFCK1 *vs*. REFCK3 groups had 21 common DEGs (**Figure 5d**). There were 16 genes that showed the same changes, in which five genes including *Prdx6*, three *GST*, and *5-OPase* were upregulated 2.54–14.17-fold and had a higher expression after a 3-day exposure, and 11 genes were downregulated −10.57- to −1.41-fold. The other five genes including three *GST*, *GT*, and *Grx* differed in variation in both groups and were upregulated after a 2-day exposure. REF201 *vs*. REF202 and REF201 *vs*. REF203 shared 12 DEGs (**Figure 5e**). There were 10 downregulated genes that had consistency changes with the higher expression levels after a 2-day exposure. The other two *GST* were found to have a reverse trend which were upregulated after a 2-day exposure. Furthermore, 11 shared DEGs were found in both the REF501 *vs*. REF502 and REF501 *vs*. REF503 groups (**Figure 5f**). Seven gene variations were consistent and downregulated −14.54- to −1.22-fold with lower expression levels after 3 days. The other four genes *GCLC*, *GHL*, *G6PD*, and *6PGD* showed reverse changes in both groups, being upregulated 1.87–4.13-fold after 2 days.

### 3.8 Transcriptomic responses of shoots and roots

**Figure 6:**
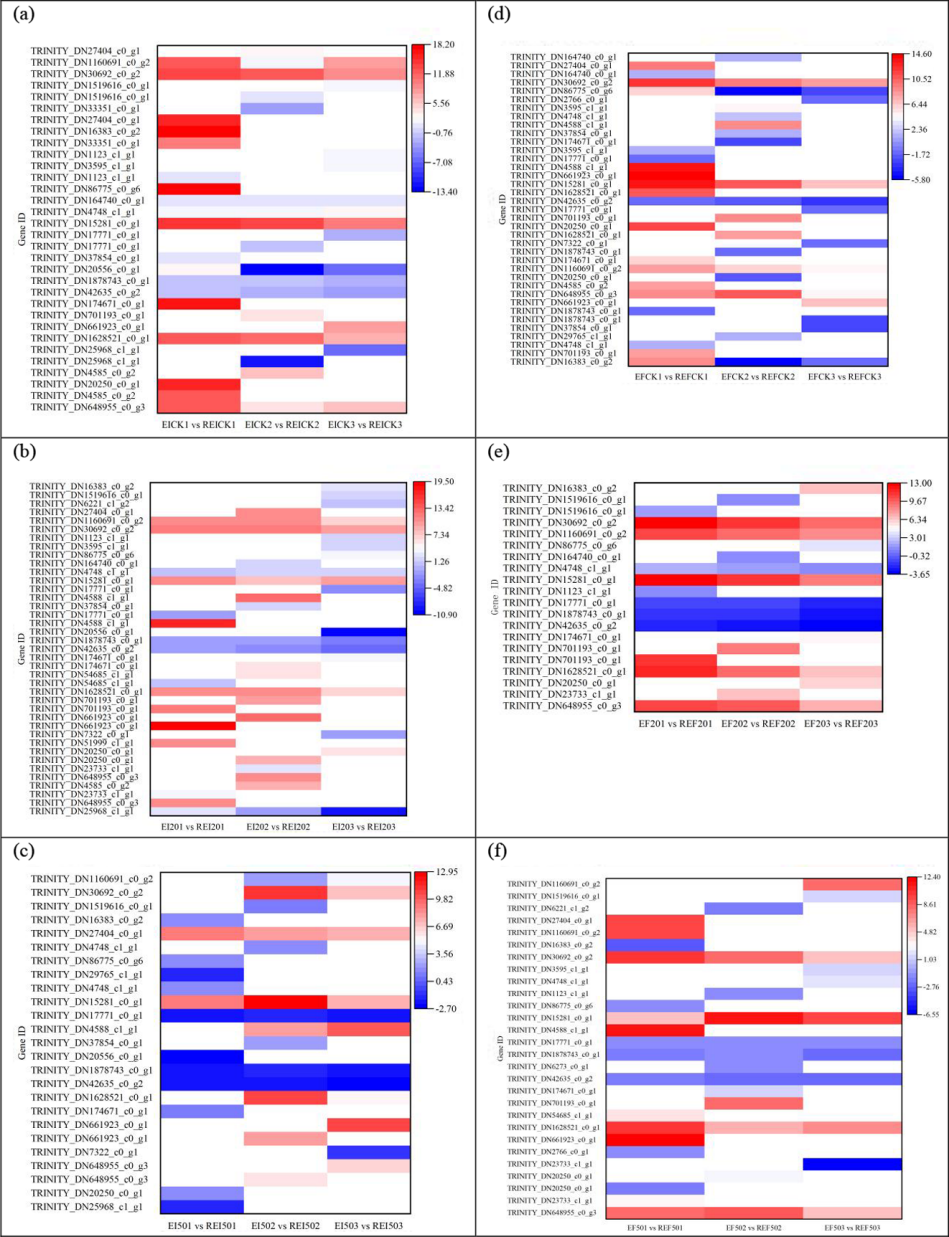
Differentially expressed genes associated with glutathione metabolism in shoots *vs*. root, showing both downregulation and upregulation. **(a)** EICK1 *vs*. REICK1, EICK2 *vs*. REICK2, and EICK3 *vs*. REICK3; **(b)** EI201 *vs*. REI201, EI202 *vs*. REI202, and EI203 *vs*. REI203; **(c)** EI501 *vs*. REI501, EI502 *vs*. REI502, and EI503 *vs*. REI503; **(d)** EFCK1 *vs*. REFCK1, EFCK2 *vs*. REFCK2, and EFCK3 *vs*. REFCK3; **(e)** EF201 *vs*. REF201, EF202 *vs*. REF202, and EF203 *vs*. REF203; and **(f)** EF501 *vs*. REF501, EF502 *vs*. REF502, and EF503 *vs*. REF503. Red and blue represent upregulated and downregulated expressions, respectively, whereas white represents no expression. Abbreviations of the samples are the same as in **Figure 1**.

Transcriptome analysis showed that the number of upregulated DEGs was much larger in the root than in the shoot (**Supplementary Table 2**; **Figure 6**). The expression levels of 20 DEGs changed (17 upregulated and 3 downregulated) in the EI202 *vs*. REI202 and EFCK1 *vs*. REFCK1 groups, whereas the expression levels of only 12 DEGs changed in the EI503 *vs*. REI503 and EF503 *vs*. REF503 samples (eight upregulated and four downregulated). In the EF202 *vs*. REF202 and EF203 *vs*. REF203 groups, there were 13 DEGs with 10 upregulated and 3 downregulated. In the EI501 *vs*. REI501 and EF502 *vs*. REF502 samples, seven upregulated and six downregulated were identified. Additionally, a total of 19, 19, 18, 17, 17, 16, 16, 14, 13, and 12 DEGs were identified in the comparisons EICK1 *vs*. REICK1, EI203 *vs*. REI203, EFCK2 *vs*. REFCK2, EICK2 *vs*. REICK2, EF501 *vs*. REF501, EICK3 *vs*. REICK3, EI201 *vs*. REI201, EI502 *vs*. REI502, EFCK3 *vs*. REFCK3, and EF201 *vs*. REF201, respectively.

A total of 10 DEGs were common to the three control EI comparisons EICK1 *vs*. REICK1, EICK2 *vs*. REICK2, and EICK3 *vs*. REICK3 (**Figure 6a**), and seven DEGs were common to the control EF comparisons EFCK1 *vs*. REFCK1, EFCK2 *vs*. REFCK2, and EFCK3 *vs*. REFCK3. These control six comparisons shared five DEGs (**Figure 6d**), such as *GCLC*, *G6PD*, two *6PGD*, and *APX*. The four former DEGs were upregulated 2.28–14.50-fold (log2), whereas *APX* was downregulated −3.48 to −1.45-fold (log2).

The three EI comparisons (EI201 *vs*. REI201, EI202 *vs*. REI202, and EI203 *vs*. REI203) under 20 mg/L Se conditions shared eight DEGs (**Figure 6b**), including *GCLC*, *GGT*, two *APX*, two *G6PD*, and two *6PGD*. The three groups showed increased expressions of *GGT*, two *G6PD*, and two *6PGD* with 1.23–12.23-fold (log2). However, two *APX* were downregulated to −4.32 to −1.58-fold and −3.34 to −1.40-fold, respectively. Five DEGs were common to the comparisons EI501 *vs*. REI501, EI502 *vs*. REI502, and EI503 *vs*. REI503 (**Figure 6c**), three *APX* reduced −2.68 to −1.24-fold, and two *6PGD* increased 7.43–12.90- and 7.57–9.19-fold (log2), respectively.

We observed nine and seven shared DEGs in the EF comparisons with supplementation of 20 and 50 mg/L Se, respectively (**Figures 6e, f**). When EF exposure to 20 mg/L Se, *GCLC*, *GGT*, *G6PD*, and two *6PGD* were upregulated 6.90–10.58-, 6.88–11.58-, 9.25–12.58-, 8.29–10.63-fold (log2), respectively, whereas two *APX* as well as *IDH* were downregulated −3.64 to −1.47 and −2.20 to -1.07-fold (log2), respectively. When EF was supplied with 50 mg/L Se, *GCLC*, *GGT*, *G6PD*, and *6PGD* were 5.03–8.66-, 5.70–9.95-, 5.01–11.38-, 4.91–9.96- up-expressed, respectively, and the two *APX* and *IDH* were −2.29 to −1.39 and −1.41 to −1.10-fold (log2) down-expressed, respectively.

## 4 Discussion

*Epichloë* species are beneficial plant endophytes that inhabit the aboveground tissues of their grass hosts, enhancing the host’s resistance to environmental stressors (Bharadwaj et al., 2020; Zamani et al., 2024). Reports have indicated that endophyte-enhanced persistence of *F. sinensis* occurs under metal stress (Wang et al., 2021a). Our study examined the glutathione redox cycle and conducted transcriptional profiling of glutathione metabolism in *F. sinensis*, both with and without *Epichloë*, under control and selenium-treated conditions.

### 4.1 Se and microorganisms affect the glutathione redox cycle

Abiotic stresses generally trigger the glutathione redox cycle in plants (Bisht and Garg, 2024). Elevated activities of GR and the GSH/GSSG ratio are vital markers for maintaining redox homeostasis (Jung et al., 2019; Adhikari et al., 2020; Wu et al., 2023). The GR activity in quinoa plants increases after 30 days of Se treatment, correlating with higher Se concentrations (2.5–20 mg/L) (Khalofah et al., 2021). Se, at specific concentrations, is known to enhance the activities of GPX and GR (Jiang et al., 2016). According to Stewart et al. (1999), the activities of SOD, CAT, APX, and GR vary with different Se concentrations, indicating that the effects of Se are dose-dependent. Consistent with this, our results indicated that the GR activity in the shoots and roots of EI and EF was enhanced by Se at lower concentrations on the first or second day. Endophyte infection was beneficial for GR, which contradicts the findings reported by Durán et al. (2016), who showed that lettuce inoculated with endophytic selenobacteria exhibited decreased GR and APX activities under Se treatment. Recently, it has been reported that mycorrhizal inoculations can upregulate GR activity in plants under metal stress (Bisht and Garg, 2024; Wu et al., 2023; Shah et al., 2024), which aligns with our findings. Furthermore, *Epichloë* spp. have been shown to produce more GR in Arizona fescue under osmotic potentials of −0.5 MPa, although they affect GR differently in sleepy grass under osmotic potentials of −0.5 and −1.7 MPa (Hamilton and Bauerle, 2012). Perhaps increased GR activity leads to the production of reactive oxygen species (ROS), resulting in increased tolerance in these host plants.

GSH is involved in the reduction of selenite and facilitates its absorption of selenite in rice roots (Zhang et al., 2015). At higher doses (25 and 50 μmol/L Se), leaf GSH levels in tomato plants increased by three to five times (Schiavon et al., 2013). Se increased the GSH levels in rice exposed to arsenic (Kumar et al., 2014). Our results indicated that GSH levels decreased in EI plants in the presence of Se, as well as in EF shoots at higher Se levels. The trend in GSH content was unstable. On the first day, the GSH levels in EI roots treated with Se were lower than those in the control group. However, on the second day, the GSH levels in both EI and EF roots were higher than in the control. Selenate treatment reduced the GSH contents in *Arabidopsis* and broccoli roots in a dose-dependent manner (Takahashi et al., 2000; Cardoso et al., 2023). The levels of GSH and GSSG in the Se-tolerant *Stanleya pinnata* and the Se-sensitive *S. albescens* showed significant decreases in the presence of 20 μmol/L selenate. The ratio of GSH to GSSG significantly dropped in the former plant, whereas it remained unaffected in the latter (Freeman et al., 2010). In the present study, both concentration- and time-dependent Se treatment experiments demonstrated that GSSG concentrations in EF shoots and roots increased after 3 days of Se treatment, whereas GSSG levels in EI shoots decreased on the third day. Additionally, higher selenium levels reduced the GSH/GSSG ratio in EF shoots, whereas Se increased the GSH/GSSG ratio in EI and EF roots on the second day. Furthermore, *Epichloë sinensis* had a significant (*p* < 0.05) effect on the glutathione redox cycle in the host (**Table 3**–6). *Epichloë sinensis* increased GSSG levels in both shoots and roots at 20 mg/L Se, as well as the GSH level and GSH/GSSG ratio in shoots, whereas it decreased GSSG levels under both control and 50 mg/L Se conditions, as well as GSH and the GSH/GSSG ratio in roots on the first day. The inoculation of arbuscular mycorrhizal fungi (AMF) significantly enhanced the GSH levels and the GSH/GSSG ratio in the roots and leaves of white clover plants under aluminum stress, as well as the root GSSG levels under aluminum stress (Khalofah et al., 2021). Additionally, AMF treatment significantly improved the GSH levels in *Brassica napus* under both control and lead stress conditions (Shah et al., 2024). Bisht and Garg (2024) also observed that mycorrhizal inoculations upregulated the GSH/GSSG ratio and total GSH levels in pigeon pea plants under cadmium stress. *Epichloë gansuensis* increased the GSH levels in *Achnatherum inebrians* plants at both 15% and 30% soil moisture but reduced the GSH levels at 60% soil moisture (Nie et al., 2024). These findings demonstrate that shifts in the glutathione redox state are influenced by various factors, including metal concentration, duration of stress, microorganism, and plant tissue and species.

### 4.2 Se and microorganisms affect glutathione metabolizing genes

GSH is important as a cofactor for glutathione peroxidase and can also activate 6-phosphogluconate dehydrogenase, thus promoting the metabolism of carbohydrate and protein (Li et al., 2019). The mechanism of Se tolerance and accumulation in certain plants may be attributed to their ability to reduce or prevent Se-related oxidative stress (Freeman et al., 2010). Both biotic and abiotic stress factors lead to variations in the expression of genes involved in GSH metabolism, including glutathione S-transferase, glutathione synthase, S-formylglutathione hydrolase, glutaredoxin, and glutathione peroxidase, as demonstrated through transcriptomic approaches (Mazumdar and Chattopadhyay, 2025; Cao et al., 2022; Yang et al., 2019; Dubey et al., 2016). A greater number of DEGs were identified in the roots compared with the shoots (**Supplementary Table 2**), reflecting the stimuli imposed by selenite as it was transported within root cells (Cao et al., 2018). Previous research has indicated that selenite treatment upregulates genes associated with glutathione metabolism in *Arabidopsis* and *Lolium perenne* (Tamaoki et al., 2008; Byrne et al., 2010). Comparative transcriptome analysis of two *Aegilops tauschii* genotypes treated with selenite revealed that several key pathways—such as phenylpropanoid biosynthesis (ko00940), carbon metabolism (ko01200), fatty acid metabolism (ko01212), and plant–pathogen interaction (ko04626)—may be involved in Se metabolism. Additionally, glutathione metabolism was uniquely identified in the comparison between selenite-treated and water-treated *A. tauschii* AS2407, but not in the corresponding comparison for AS65 (Wu et al., 2019). The loss of function of *APX*1 in *Arabidopsis thaliana* resulted in tolerance to Se stress (Jiang et al., 2016). In this study, the most common highly enriched metabolic pathways were carbon metabolism, phenylpropanoid biosynthesis, biosynthesis of amino acids (ko01230), oxidative phosphorylation (ko00190), starch and sucrose metabolism (ko00500), plant–pathogen interaction, ABC transporters (ko02010), MAPK signaling pathway (ko04016), plant hormone signal transduction (ko04075), phosphatidylinositol signaling system (ko04070), etc. A greater number of DEGs involved in glutathione metabolism were significantly upregulated in the shoots of EI and EF plants grown subjected to selenite treatment (**Figure 1**), including those encoding *GST* and *GR*, with the highest abundance observed at the elevated Se levels, potentially contributing to selenite. Comparisons between shoots and roots showed that most genes related to glutathione synthesis had higher expression in roots, but *APX* genes showed lower expression levels, implying that the root is among the first organs to sense unfavorable conditions. Additionally, *GST* genes were expressed in the roots (**Figure 4**), which are considered to be Se-responsive genes in various plant species (Freeman et al., 2010; Tamaoki et al., 2008). Following long-term selenite treatment of tea seedlings, 169 of upregulated genes in the roots were enriched in GSH metabolism, encoding *GST*, *GR*, and *GPX* (Cao et al., 2018). Similarly, after 48 h of treatment with 5 μmol/L selenite, DEGs related to *GST* and *APX* were upregulated in tea roots (Ren et al., 2022). Several *GST* genes were significantly expressed in both tea roots and leaves, and the highest abundance existed in roots rather than leaves (Cao et al., 2018). The positive effect of *GST* in enhancing stress tolerance have been emphasized in plants (Brentner et al., 2008; Xu et al., 2016; Wang et al., 2019), which may be suggesting their detoxification function (Ren et al., 2022). Furthermore, our results indicated that expression of the *GCLC* genes was higher in the roots than in the shoots (**Figure 6**). Hence, genes related to glutathione metabolism may be involved in Se uptake in roots. After long-term or short-term selenite treatment in tea, the expression of glutathione metabolizing genes was differentially regulated (Ren et al., 2022; Cao et al., 2018), with a notable trend of downregulation of gene as the duration of treatment increased in this study. The number of genes regulating other metabolite biosynthesis will be the potential candidates for Se-related function analysis in our future study.

Arbuscular mycorrhizal fungi and *Epichloë* species increased the contents of GSH and GR in plants subjected to adverse environmental stresses by upregulating the expression of plant genes such as *GPX*, *GSH2*, *GS*, and *GSH1*, which are associated with antioxidant biosynthetic enzymes (Nie et al., 2024; Li et al., 2022; Ye et al., 2019). Endophyte-infected tall fescue under water stress upregulated the expression of *GST* genes but repressed their expression of these genes under non-stress conditions (Dinkins et al., 2019). The *Epichloë gansuensis* enhanced the expression of *GPX*, *GSH*, and *APX* genes in hosts exposed to low soil moisture, while reducing the expression of most *GST* genes in hosts with adequate moisture (Nie et al., 2024). The application of mycorrhizal fungi with selenium influenced the expression of genes related to glutathione metabolism in rice roots (Qin et al., 2023). In this context, DEGs related to ko00940, ko01230, ko00190, ko02010, ko04016, and ko04075 were also enriched for the EF *vs*. EI group. Consistent with the accumulation of GR in shoots, endophyte colonization enhanced the expression of *GR* (**Supplementary Figure 2**). Moreover, the endophyte increased the expression of *GCLC* and *APX*, in shoots in response to 20 mg/L Se and of *Grx* on the second day in the presence of Se, while reducing the expression of genes encoding *GST* (**Figure 3**). These results may indicate the protective role of *Epichloë* in plant by reducing toxic reactive species under Se stress. Analysis of these DEGs revealed that *Epichloë sinensis* and Se concentration regulated glutathione metabolism during the time period. *Epichloë sinensis* is more conducive to maintaining high levels of glutathione by increasing the expression of *GR* genes and reducing the expression of *GST* genes.

## 5 Conclusions

The present study integrated physiological and transcriptomic analyses to elucidate the influence of Se treatment and *Epichloë* endophyte infection on glutathione metabolism in *F. sinensis* shoots and roots. The *Epichloë* endophyte or Se concentration significantly affected the glutathione reductase, reduced glutathione, oxidized glutathione, and the GSH/GSSG ratio over time. A significant number of genes involved in glutathione metabolism in the EI plant were upregulated in response to Se, encoding glutathione S-transferase and glutathione reductase. Therefore, *Epichloë* endophyte is suggested as a tool for the production of Se-rich forage. Further functional studies on glutathione metabolic genes in *F. sinensis* infected with *Epichloë sinensis* associations exposed to Se should be conducted in the future.

## Data Availability

The datasets used and/or analyzed during the current study are available within the article. The RNA-seq data is deposited in the NCBI Sequence Read Archive (SRA) database under accession number PRJNA1358410.
