# Supplementary material for: Transcriptional profiling of glutathione metabolism and the glutathione redox cycle of Festuca sinensis infected with Epichloë sinensis in response to Na2SeO3
Source: Front Plant Sci. 2026 Mar 19;17:1752324. doi: 10.3389/fpls.2026.1752324 (PMC13044120; doi:10.3389/fpls.2026.1752324)
Supplement: Supplementary file 1 [file DataSheet1.docx]

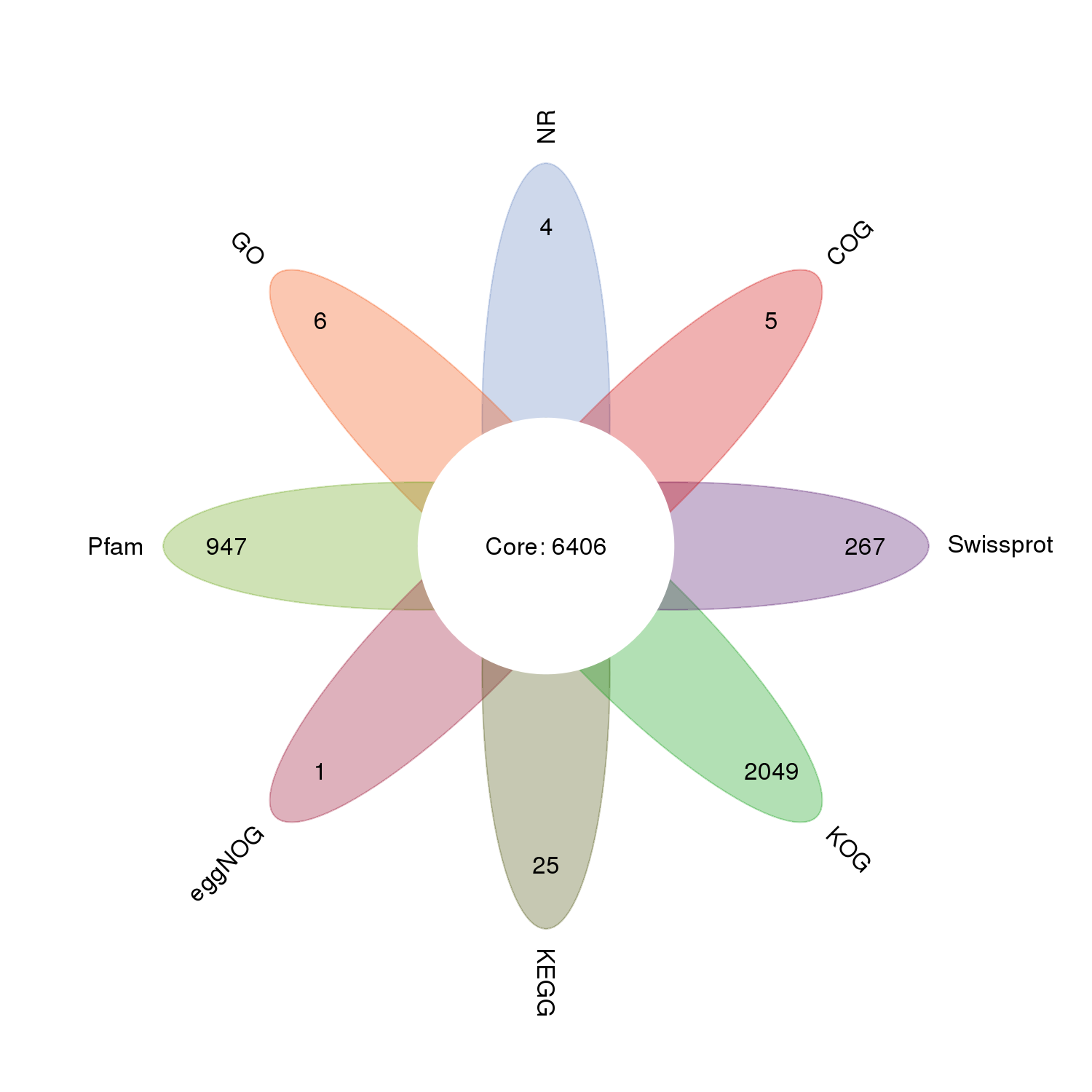


Figure S1 Numbers of unigene annotated within the eight different public databases.

Table S1 Number of up- and down-regulated differently expressed unigenes in comparison groups.

| Group | Up DEGs | Down DEGs | Group | Up DEGs | Down DEGs | Group | Up DEGs | Down DEGs |
| --- | --- | --- | --- | --- | --- | --- | --- | --- |
| Shoot group | | | | | | | | |
| EICK1 vs EI201 | 5894 | 337 | EICK2 vs EI202 | 2400 | 1330 | EICK3 vs EI203 | 1656 | 358 |
| EICK1 vs EI501 | 5566 | 395 | EICK2 vs EI502 | 3603 | 1631 | EICK3 vs EI503 | 1294 | 761 |
| EFCK1 vs EF201 | 1719 | 793 | EFCK2 vs EF202 | 273 | 4755 | EFCK3 vs EF203 | 835 | 4469 |
| EFCK1 vs EF501 | 6025 | 602 | EFCK2 vs EF502 | 1505 | 2417 | EFCK3 vs EF503 | 2499 | 4648 |
| EICK1 vs EICK2 | 4180 | 471 | EI201 vs EI202 | 2034 | 3639 | EI501 vs EI502 | 3988 | 4105 |
| EICK1 vs EICK3 | 1116 | 873 | EI201 vs EI203 | 2485 | 6417 | EI501 vs EI503 | 2292 | 5820 |
| EFCK1 vs EFCK2 | 4927 | 793 | EF201 vs EF202 | 465 | 1304 | EF501 vs EF502 | 637 | 3109 |
| EFCK1 vs EFCK3 | 5400 | 1523 | EF201 vs EF203 | 957 | 1629 | EF501 vs EF503 | 1735 | 5833 |
| EF vs EI | | | | | | | | |
| EFCK1 vs EICK1 | 1797 | 784 | EF201 vs EI201 | 5751 | 825 | EF501 vs EI501 | 4662 | 3715 |
| EFCK2 vs EICK2 | 3711 | 4403 | EF202 vs EI202 | 5433 | 525 | EF502 vs EI502 | 6391 | 3990 |
| EFCK3 vs EICK3 | 2240 | 4994 | EF203 vs EI203 | 2720 | 695 | EF503 vs EI503 | 2445 | 2543 |
| Root group | | | | | | | | |
| REICK1 vs REI201 | 6715 | 4889 | REICK2 vs REI202 | 8085 | 1911 | REICK3 vs REI203 | 3844 | 3704 |
| REICK1 vs REI501 | 3242 | 2895 | REICK2 vs REI502 | 8643 | 2414 | REICK3 vs REI503 | 5421 | 2825 |
| REFCK1 vs REF201 | 3845 | 5369 | REFCK2 vs REF202 | 1141 | 6179 | REFCK3 vs REF203 | 2602 | 5656 |
| REFCK1 vs REF501 | 8097 | 2590 | REFCK2 vs REF502 | 3952 | 5098 | REFCK3 vs REF503 | 2101 | 6621 |
| REICK1 vs REICK2 | 1876 | 5278 | REI201 vs REI202 | 6721 | 6445 | REI501 vs REI502 | 5696 | 4013 |
| REICK1 vs REICK3 | 5055 | 6905 | REI201 vs REI203 | 5558 | 9366 | REI501 vs REI503 | 4913 | 5955 |
| REFCK1 vs REFCK2 | 5578 | 3746 | REF201 vs REF202 | 1592 | 3199 | REF501 vs REF502 | 3637 | 8279 |
| REFCK1 vs REFCK3 | 5164 | 5438 | REF201 vs REF203 | 4186 | 5115 | REF501 vs REF503 | 2346 | 11334 |
| Shoot vs Root | | | | | | | | |
| EICK1 vs REICK1 | 7162 | 1183 | EI201 vs REI201 | 6602 | 2256 | EI501 vs REI501 | 3144 | 3113 |
| EICK2 vs REICK2 | 4739 | 4690 | EI202 vs REI202 | 9401 | 4102 | EI502 vs REI502 | 6602 | 2109 |
| EICK3 vs REICK3 | 6783 | 3371 | EI203 vs REI203 | 5871 | 4179 | EI503 vs REI503 | 6237 | 1931 |
| EFCK1 vs REFCK1 | 8075 | 1771 | EF201 vs REF201 | 5733 | 2299 | EF501 vs REF501 | 7479 | 2395 |
| EFCK2 vs REFCK2 | 6471 | 3703 | EF202 vs REF202 | 4582 | 1367 | EF502 vs REF502 | 3758 | 2326 |
| EFCK3 vs REFCK3 | 4008 | 2523 | EF203 vs REF203 | 5119 | 2365 | EF503 vs REF503 | 2757 | 3385 |

Note: EICK1 vs EI201 represents the comparison of DEGs in the shoot of endophyte-infected *F. sinensis* supplied without and with 20 mg/L Na_2_SeO_3_ on day 1; EICK1 vs EI501 represents the comparison of DEGs in the shoot of endophyte-infected *F. sinensis* supplied without and with 50 mg/L Na_2_SeO_3_ on day 1; EICK2 vs EI202 represents the comparison of DEGs in the shoot of endophyte-infected *F. sinensis* supplied without and with 20 mg/L Na_2_SeO_3_ on day 2; EICK2 vs EI502 represents the comparison of DEGs in the shoot of endophyte -infected *F. sinensis* supplied without and with 50 mg/L Na_2_SeO_3_ on day 2; EICK3 vs EI203 represents the comparison of DEGs in the shoot of endophyte-infected *F. sinensis* supplied without and with 20 mg/L Na_2_SeO_3_ on day 3; EICK3 vs EI503 represents the comparison of DEGs in the shoot of endophyte-infected *F. sinensis* supplied without and with 50 mg/L Na_2_SeO_3_ on day 3; EFCK1 vs EF201 represents the comparison of DEGs in the shoot of endophyte-free *F. sinensis* supplied without and with 20 mg/L Na_2_SeO_3_ on day 1; EFCK1 vs EF501 represents the comparison of DEGs in the shoot of endophyte-free *F. sinensis* supplied without and with 50 mg/L Na_2_SeO_3_ on day 1; REICK1 vs REI201 represents the comparison of DEGs in the root of endophyte-infected *F. sinensis* supplied without and with 20 mg/L Na_2_SeO_3_ on day 1. And so on.

up

Cys +Gly

EC:3.4.13.-

5-oxoprolinase (*5-OPase*)

Glutamate

Glutamate

Cys-gly + Oxoproline

γ-glutamylcyclotranspeptidase (*GGCT*)

γ-glutamyltranspeptidase (*GGT*)

Glutathione S-transferase (*GST*)

R-S-Glutathione

Ascorbate peroxidase (*APX*)

Dehydroascorbate

Glutathionyl spermidine

Glutathione synthase (*GS*)

γ-Glutammyl-cysteine

Glutathione peroxidase (*GPX*)

Glutathione (GSH)

Glutathione reductase (*GR*)

Glutathione disulfide (GSSD)

down

Figure S2 GSH recycling in plants, and significance of differentially expressed genes in shoot EF vs EI groups.

Table S2 Changes in differentially expressed genes associated with glutathione metabolism based on KEGG analysis between different groups

| Group | Up DEGs | Down DEGs | Group | Up DEGs | Down DEGs | Group | Up DEGs | Down DEGs |
| --- | --- | --- | --- | --- | --- | --- | --- | --- |
| Shoot group | | | | | | | | |
| EICK1 vs EI201 | 27 | 1 | EICK2 vs EI202 | 10 | 16 | EICK3 vs EI203 | 9 | 0 |
| EICK1 vs EI501 | 19 | 1 | EICK2 vs EI502 | 15 | 7 | EICK3 vs EI503 | 9 | 0 |
| EFCK1 vs EF201 | 3 | 2 | EFCK2 vs EF202 | 6 | 18 | EFCK3 vs EF203 | 1 | 21 |
| EFCK1 vs EF501 | 31 | 1 | EFCK2 vs EF502 | 12 | 9 | EFCK3 vs EF503 | 17 | 20 |
| EICK1 vs EICK2 | 19 | 2 | EI201 vs EI202 | 3 | 21 | EI501 vs EI502 | 18 | 18 |
| EICK1 vs EICK3 | 5 | 3 | EI201 vs EI203 | 13 | 26 | EI501 vs EI503 | 9 | 17 |
| EFCK1 vs EFCK2 | 21 | 8 | EF201 vs EF202 | 3 | 5 | EF501 vs EF502 | 2 | 20 |
| EFCK1 vs EFCK3 | 29 | 5 | EF201 vs EF203 | 5 | 4 | EF501 vs EF503 | 16 | 25 |
| EF vs EI | | | | | | | | |
| EFCK1 vs EICK1 | 5 | 6 | EF201 vs EI201 | 22 | 7 | EF501 vs EI501 | 16 | 37 |
| EFCK2 vs EICK2 | 18 | 19 | EF202 vs EI202 | 14 | 12 | EF502 vs EI502 | 22 | 20 |
| EFCK3 vs EICK3 | 6 | 34 | EF203 vs EI203 | 5 | 3 | EF503 vs EI503 | 5 | 22 |
| Root group | | | | | | | | |
| REICK1 vs REI201 | 35 | 21 | REICK2 vs REI202 | 33 | 7 | REICK3 vs REI203 | 25 | 10 |
| REICK1 vs REI501 | 13 | 15 | REICK2 vs REI502 | 34 | 11 | REICK3 vs REI503 | 24 | 18 |
| REFCK1 vs REF201 | 21 | 26 | REFCK2 vs REF202 | 7 | 26 | REFCK3 vs REF203 | 33 | 19 |
| REFCK1 vs REF501 | 31 | 27 | REFCK2 vs REF502 | 16 | 23 | REFCK3 vs REF503 | 32 | 22 |
| REICK1 vs REICK2 | 8 | 22 | REI201 vs REI202 | 36 | 22 | REI501 vs REI502 | 25 | 17 |
| REICK1 vs REICK3 | 22 | 29 | REI201 vs REI203 | 28 | 38 | REI501 vs REI503 | 8 | 14 |
| REFCK1 vs REFCK2 | 26 | 14 | REF201 vs REF202 | 15 | 9 | REF501 vs REF502 | 4 | 9 |
| REFCK1 vs REFCK3 | 13 | 46 | REF201 vs REF203 | 12 | 28 | REF501 vs REF503 | 5 | 16 |
| Shoot vs Root | | | | | | | | |
| EICK1 vs REICK1 | 17 | 2 | EI201 vs REI201 | 13 | 3 | EI501 vs REI501 | 7 | 6 |
| EICK2 vs REICK2 | 11 | 6 | EI202 vs REI202 | 17 | 3 | EI502 vs REI502 | 11 | 3 |
| EICK3 vs REICK3 | 11 | 5 | EI203 vs REI203 | 13 | 6 | EI503 vs REI503 | 8 | 4 |
| EFCK1 vs REFCK1 | 17 | 3 | EF201 vs REF201 | 9 | 3 | EF501 vs REF501 | 10 | 7 |
| EFCK2 vs REFCK2 | 12 | 6 | EF202 vs REF202 | 10 | 3 | EF502 vs REF502 | 7 | 6 |
| EFCK3 vs REFCK3 | 5 | 8 | EF203 vs REF203 | 10 | 3 | EF503 vs REF503 | 8 | 4 |
